# An Ethnobotanical Survey and Pharmacological and Toxicity Review of Medicinal Plants Used in the Management of Obesity in the North Central Zone of Nigeria

**DOI:** 10.1155/jobe/5568216

**Published:** 2025-02-05

**Authors:** Gabriel O. Anyanwu, Dorathy Anzaku, Yanga J. Bulus, Jemimah N. Girgi, Chinda C. Donwell, Jerome O. Ihuma, Eusebius C. Onyeneke, Giovanna Bermano, Vanessa Steenkamp

**Affiliations:** ^1^Department of Biochemistry, Bingham University, Karu, Nasarawa, Nigeria; ^2^Department of Biological Sciences, Bingham University, Karu, Nasarawa, Nigeria; ^3^Department of Biochemistry, University of Benin, Benin City, Edo State, Nigeria; ^4^Centre for Obesity Research and Education (CORE), School of Pharmacy, Applied Sciences and Public Health, Robert Gordon University, Aberdeen, UK; ^5^Department of Pharmacology, Faculty of Health Sciences, University of Pretoria, Pretoria, South Africa

**Keywords:** medicinal plants, Nigeria, obesity, pharmacological activity, survey, toxicity

## Abstract

**Introduction:** Obesity is increasing worldwide. Due to the unavailability of affordable obesity drugs in most parts of Nigeria, many overweight and obese people rely on medicinal plants to manage obesity. Thus, the aim of this study is to document medicinal plants traditionally used in the treatment and management of obesity in the North Central Zone of Nigeria, determine the plants to which pharmacological assessment of their use in obesity management has not been reported, and assess their toxicity based on the literature.

**Methods:** Semistructured questionnaires and interviews were used to assess sociodemographic information of the 700 herb sellers/practitioners (100 for each state) who consented to participate in the study. Information gathered on plants that are traditionally used in the management of obesity included administration/dosage, method of preparation, plant part used, method of growth, and plant type. The field study was conducted over a one-year period, from March 2018 to March 2019. Reports of pharmacological activity pertaining to obesity as well as toxicity of the plants were obtained from the literature via scientific databases (Scopus, Web of Science, PubMed, Google Scholar, SciFinder, AJOL, PubChem, and other web sources) after the field survey.

**Results:** A total of 39 families and 70 plant species were used to treat or manage obesity. The majority of plant species used resulted in the family Leguminosae. The relative frequency of citation (RFC) and percentage values for the five most frequently used plants were as follows: *Citrus aurantifolia* (0.0500; 3.56%), *Citrus limon* (0.0457; 3.26%), *Garcinia kola* (0.0429; 3.05%), *Zingiber officinale* (0.0429; 3.05%), and *Allium sativum* (0.0414; 2.95%). The majority of the medications were prepared as decoctions (50.5%), and cultivated plants (62.86%) were in the majority of plants used. Results showed that 23 plants have no pharmacological report for antiobesity activities while among the five frequently used plants, only *Garcinia kola* was reported toxic in preclinical models.

**Conclusions:** This paper provides a valuable compilation of the plants used in obesity treatment in the study area by indigenous healers, highlights plants with no reported pharmacological activity pertaining to obesity, and indicates the toxicity profile of used plants. However, further studies on the mechanism of action are warranted, especially where no reports were obtained.

## 1. Introduction

Obesity is an increasing public health problem in Africa [1]. Although undernutrition is still persistent in Africa, there is a rising prevalence of overnutrition, especially in children younger than 5 years of age [2] and people older than 18 years [3]. Obesity is defined as the disproportionate accumulation of adipose tissue that is detrimental to a person's health [4]. This may result in the development of insulin resistance, type 2 diabetes mellitus, heart-related diseases, osteoarthritis, and sleep apnea [5–7].

In Africa, overweight/obese children doubled in number, increasing from 5.4 million in 1990 to 10.3 million in 2014 [8]. Globally, over 650 million adults were reported to be obese [9] and more than 1.9 billion overweight [8]. Furthermore, overweight/obese children and adolescents (aged 5–19) accounted for 340 million incidences in the same year [8].

National data on obesity trends in Nigeria are scarce, and existing studies report obesity prevalence only for pockets of Nigeria's population. In Maiduguri, the capital of Borno State in North-East Nigeria, the prevalence of obesity was reported as 8.1% [10], compared to 22.2% in Lagos State (South-West Nigeria), the commercial hub of Nigeria [11]. Overweight individuals were reported to present between 20.3% and 35.1% in Nigeria in 2013 [12]. In the latest account by Akarolo-Anthony et al. [13], approximately two-thirds of professional and high socioeconomic status Nigerian adults living in urban cities, particularly Abuja (North-Central Nigeria), were either overweight or obese. The latter is indicative thereof that there is an increase in the prevalence of obesity over the last decade.

Factors that have led to an increase in obesity in Nigeria include urbanization, increased socioeconomic status, consumption of high-energy-density foods, and less physical activity due to mechanized transport [1, 3, 14]. An exacerbating cause of the obesity situation in Africa is the antiquated traditional African conception of affluence, which connotes that obesity in women is an indicator of the family's material abundance [15]. In the past, Nigerian women were less concerned about their weight; in fact, skinny young women were made fat before they were allowed to marry their husbands [16]. Increased awareness regarding the health consequences of obesity across the country appears to be gradually eroding the cultural belief that obesity is associated with affluence or is indicative of good living [13, 17].

People who are overweight or obese are consistently searching for orthodox or traditional medicines which can be used to reduce weight. Orthodox medications for the treatment and management of obesity such as orlistat, liraglutide, lorcaserin, naltrexone–bupropion, and phentermine–topiramate are available [4, 18]; however, these drugs are very expensive and not affordable to the majority of people living in African countries. This has led to many seeking alternatives in the form of traditional or herbal medicines [19].

The documentation of medicinal plants used in the herbal management of obesity has been attempted in the South West Zone of Nigeria, particularly Lagos State [20, 21] and also in the Nomad and Hunter Communities of Burkina Faso [15]. However, these studies were not comprehensive and limited in their objectives. Thus, the aim of the study was to document medicinal plants used by herbal practitioners and herb sellers in the North Central and West Zones of Nigeria for the management of obesity. The objectives include (i) to ascertain the demographic characteristics of herb sellers and practitioners for the management of obesity in the study area, (ii) to identify and document the medicinal plants used for the traditional management of obesity by herbal practitioners and herb sellers in the study area, (iii) to identify the most common and popularly used medicinal plants for the treatment and management of obesity in the study area, (iv) to determine the plants to which pharmacological assessment of their use in obesity management has not been reported based on the literature, and (v) to assess the toxicity of identified medicinal plants used for the treatment and management of obesity in the study area based on the literature.

This study is significant as it would update the current knowledge of medicinal plants used traditionally in the management of obesity in Northern Nigeria and by extension in Africa. It would identify the plants whose antiobesity properties have not been reported among the plants identified to be used traditionally to treat or manage obesity. The study presents comprehensive work done on the traditional management of obesity using herbs including identifying the toxicity profile of the plants with which readers could draw an inference as to the plants that require further studies for their antiobesity and toxicological studies.

## 2. Materials and Methods

### 2.1. Study Area

Nigeria is a country located in West Africa and covers a total area of 923,768 km^2^ (356,669 sq mi). It shares borders with Niger in the north, Chad and Cameroon in the east, and Benin in the west. Nigeria is a large country, extending between latitudes 4° and 14°N, and longitudes 2° and 15°E. It consists of 36 states and the Federal Capital Territory (FCT), Abuja. These states are grouped into six geopolitical zones based on similar history, cultures, languages and close territories, and also for administrative purposes. The 6 geopolitical zones are North West (7 States), North Central (7 States), North East (6 States), South West (6 States), South-South (6 States), and South East (5 States). This study was carried out in the North Central Zone states (Niger, Kwara, Kogi, Nasarawa, Plateau, Benue states, and the FCT, Abuja).

**Figure 1:**
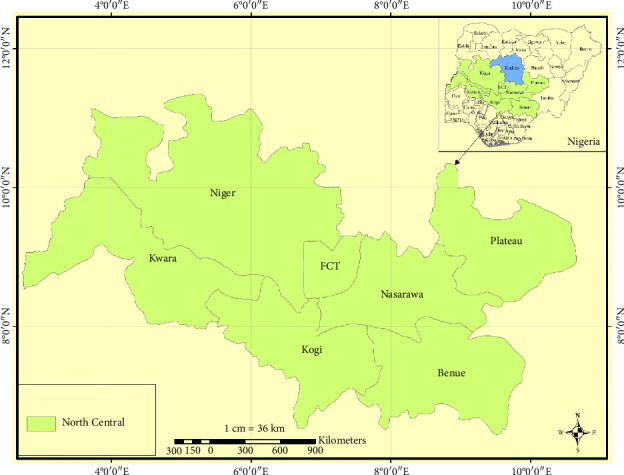
North Central Zone of Nigeria.

The seven states in the North Central Zone are situated geographically in the middle belt region of Nigeria, beginning from the west across the confluence of the River Niger and the River Benue to the east (Figure 1). The region has an abundance of natural land features, hills, highlands, and a rich agricultural region. The FCT Abuja is where the seat of the government of Nigeria is strategically located at the center of the country while Benue is often thought of as the “food basket of the nation” due to its rich agricultural activities compared to other states. The Savannas of Niger, Plateau, Nasarawa, and FCT trail into the rain forests found partly in other states like Kwara, Kogi, and Benue. The climate in the North Central is equatorial, with apparently expressed rainy seasons and an average annual temperature of about 30°C. The population of Nigeria is estimated to be 193,392,517 persons in 2016 based on the population census conducted in 2006 by the National Population Commission [22]. The population forecasts by states for states in the study area are Benue (5,741,815), FCT (3,564,126), Kogi (4,473,490), Kwara (3,192,893), Nasarawa (2,523,395), Niger (5,556,247), and Plateau (4,200,442).

### 2.2. Field Interviews

The study was conducted over a one-year period, from March 2018 to March 2019, and included field interviews with herb sellers and herbal practitioners to document medicinal plants traditionally used in the treatment of obesity or to enhance weight loss. The recruitment of participants was in some cases facilitated by village heads, ward heads, and key informants to locate herbal practitioners and sellers and involved face-to-face interactions. The objectives and interview procedure of the survey were explained to the participants in their native language and in some cases with the aid of an interpreter before verbal consent was obtained. Participants were selected randomly (irrespective of gender and age) and were only allowed to participate if verbal consent was given. Participants were interviewed using semistructured questionnaires and interview questions designed for the collection of information sought. One hundred interviews were conducted in each of the 7 states selected for this study. Sociodemographic information noted included age, gender, educational level, and trade as indicated in Table 1. With regard to the plants used in the treatment of obesity, information recorded included administration/dosage, method of preparation, plant part used, method of growth, and plant type.

### 2.3. Plant Collection and Identification

The medicinal plants used for the treatment of obesity were collected or bought from herb sellers/practitioners. The identification and authentication of the plants were carried out with the assistance of a taxonomist at the University of Agriculture, Makurdi, Benue State, Nigeria and Bingham University, Karu, Nasarawa State, Nigeria. Voucher specimens were prepared and deposited at the herbarium of the University of Agriculture. The plant names and families were verified on https://www.theplantlist.org before inclusion in the study.

### 2.4. Plant Pharmacological and Toxicological Activities

The information on plant pharmacological and toxicological activities that are associated with their antiobesity and toxicity activities was found in the literature via scientific databases (Scopus, Web of Science, PubMed, Google Scholar, SciFinder, AJOL, PubChem, and other web sources such as the Plant List, Kew Botanical Garden, and PROTA).

### 2.5. Data Analysis

The ethnobotanical survey data were analyzed using descriptive statistical methods of frequencies and percentages. Data on the frequency of citation (FC) of the plants used for obesity treatment were recorded. Data on the FC of the plants used for obesity treatment were analyzed as the relative frequency of citation (RFC) and percentage value. The relative importance of a particular plant species was determined by calculating RFC according to Tardıo and Pardo-de-Santayana [23], and the RFC = Fc/*N*, where *N* is the total number of respondents and Fc is the number of respondents who cited a particular plant species. The percentage value of plant or percent FC showed the most important plant species used for obesity management which was calculated as % value = (Fc/*n*) × 100 [24], where n is the total number of citations of all the species and Fc represented use reports or FC of a particular plant species.

## 3. Results

### 3.1. Sociodemographic Information of the Informants Interviewed

A total of 700 informants from 7 states were interviewed to collect information on traditional medicinal plants used for the treatment of obesity (Table 1). A larger proportion of men (66.1%) constituted the study population. Most of the informants fell in the age bracket of 36–59 years (58.0%). Most respondents had attained primary education (47.0%); however, no form of education was documented in 30.3% of respondents and training was obtained by relatives. Respondents were rather herb sellers than herbal practitioners (Table 1).

### 3.2. Distribution of Plant Species and Families

A total of 39 families and 70 plant species were used to treat or manage obesity in the North Central States (Table 2). The family with the highest representation in terms of the number of species is Leguminosae (10 species), followed by Cucurbitaceae (8 species). Other plant families in the study include Apocynaceae, Arecaceae, Capparaceae, Combretaceae, Cucurbitaceae, Leguminosae, Malvaceae, Meliaceae, Moringaceae, Pedaliaceae, Poaceae, Rhamnaceae, Rubiaceae, and Rutaceae.

### 3.3. Method of Preparation and Plant Parts Used

Among the various parts of the plants used for obesity treatment, the leaves of 29 plants, fruits of 21, and seeds of 14 were used most frequently (Table 2). Roots (8 plants), stem (5 plants), and stem bark (5 plants) were also used often. The most common way plants were prepared was as a decoction (50.5%), followed by juicing (21.2%), maceration (19.2%) in alcohol, and powdered form (9.1%).

### 3.4. Domestication Status and Life Forms of Medicinal Plants

Most of the recorded plants used for obesity treatment/management were cultivated (62.9%) with 14.26% found in the wild, and 22.9% representing both cultivated and found in the wild. Furthermore, most were herbs (57.1%), with 34.3% and 8.6% being trees and shrubs, respectively. A larger number of the plants are bought directly from the herbal stores or markets for the preparation of medicinal formulations, specifically 51 plants, others are acquired with the assistance of herbal practitioners, and these plants include *Adansonia digitata, Alstonia boonei, Anthocleista vogelii, Azadirachta indica, Boscia senegalensis, Ceratotheca sesamoides, Chrozophora senegalensis, Cinnamomum verum, Citrus limon, Cochlospermum tinctorium, Commiphora africana, Cymbopogon citratus, Detarium senegalensis, Guiera senegalensis, Heinsia crinita, Khaya senegalensis, Luffa aegyptiaca, Prosopis africana*, and *Ziziphus jujube*.

### 3.5. Dosage and Route of Administration

Informants referred to the quantity of prepared herb used as a cup (which meant a minimum of 250 mL), half a cup (≤ 100 mL), or a small cup (≤ 50 mL). The plants are consumed mainly through the oral route (Table 2), and external use of the prepared plants' parts such as dermal application, body bath, soaking, or steaming methods was not reported.

### 3.6. FC, RFC, and Percentage Values of Plants

The RFCs of the plants species with above 20 FCs are *Allium sativum* (0.0414), *Aloe vera* (0.0357), *Cinnamomum verum* (0.0329), *Citrus aurantifolia* (0.0500), *Citrus limon* (0.0457), *Cucumis sativus* (0.0300), *Fragaria vesca* (0.0386), *Gongronema latifolium* (0.0300), *Garcinia kola* (0.0429), *Hibiscus sabdariffa* (0.0314), *Ocimum gratissimum* (0.0286), *Pterocarpus mildbraedii* (0.0371), *Vernonia amygdalina* (0.0300), and *Zingiber officinale* (0.0429). The majority of plant species used resorted in the family Leguminosae (10 species), followed by Cucurbitaceae (8 species). The percentage values for the top five plants used were *Citrus aurantifolia* (3.56%), *Citrus limon* (3.26%), *Garcinia kola* (3.05%), *Zingiber officinale* (3.05%), and *Allium sativum* (2.95%).

### 3.7. Antiobesity Pharmacological Effects of the Plants

Plants identified for their traditional medicinal uses against obesity with no report on their antiobesity pharmacological effects in the literature include *Acacia nilotica, Beta vulgaris, Boscia senegalensis, Capsicum chinense, Ceratotheca sesamoides, Chrozophora senegalensis, Cochlospermum tinctorium, Commiphora africana, Detarium senegalensis, Fragaria vesca, Garcinia kola, Gnetum africanum, Gongronema latifolium, Guiera senegalensis, Heinsia crinita, Luffa aegyptiaca, Mentha pulegium, Momordica balsamina, Piper guineense, Prosopis africana, Senna occidentalis*, and *Vigna subterranea*.

Antiobesity activity has been reported for some of the documented plants and this has been summarized (Table 3). Some plants have been well researched for their weight loss abilities (including mechanism of action) and include *Allium cepa, Allium sativum, Aloe vera, Capsicum annuum, Cinnamomum verum, Citrullus colocynthis, Citrus aurantifolia, Citrus limon, Citrus paradise, Cocos nucifera, Cucurbita ficifolia, Curcuma longa, Glycine max, Hibiscus sabdariffa, Ipomoea batatas, Irvingia gabonensis, Momordica charantia, Moringa oleifera, Myristica fragrans, Persea americana, Piper nigrum, Psidium guajava, Spinacia oleracea, Tamarindus indica, Tetrapleura tetraptera, Vernonia amygdalina, and Zingiber officinale.*

### 3.8. Toxicity Profile of Plants

Reports on toxicity screening of the plant extracts and fractions using various in vitro and in vivo models have been summarized (Table 4). There were 61 plants that had LD_50_ values > 2000 mg/kg when administered either orally or intraperitoneally to rats and/or mice. The plants with LD_50_ values < 2000 mg/kg were fewer, and it is important to note that majority of the plants in this category were administered intraperitoneally in mice: *Commiphora africana*, *Cucurbita ficifolia*, *Cocos nucifera*, *Cochlospermum tinctorium*, *Gongronema latifolium*, *Khaya senegalensis*, *Prosopis africana*, and *Tetrapleura tetraptera*; on the other hand, oral acute toxicity studies revealed *Citrullus colocynthis* was toxic in rats, while *Garcinia kola* and *Guiera senegalensis* were toxic in mice (Table 4).

Plants that were reported as toxic in rodents in subacute toxicity studies (administration between 14 days and 30 days) can be categorized in function as doses: ≤ 300 mg/kg (*Cucurbita pepo* stem, *Citrullus colocynthis* seeds, *Azadirachta indica* bark, and *Vernonia amygdalina* leaves); 500–1000 mg/kg (*Acacia nilotica* roots, *Glycine max* oil, *Gnetum africanum* leaves, *Guiera senegalensis* roots & leaves, *Myristica fragrans* mace, *Ocimum gratissimum* leaves, *Psidium guajava* bark, *Persea americana* seeds, *Aloe vera* gel, *Ipomoea batatas* roots, *Lycopersicon esculentum* leaves, and *Moringa oleifera* leaves and seeds); and ≥ 2000 mg/kg (*Mentha pulegium* fruits).

Toxicity studies, conducted for a period between 30 and 60 days, revealed that more plants were toxic to rodents at concentrations of ≤ 500 mg/kg including *Citrullus colocynthis* fruit, *Murraya koenigii* leaves, *Citrus aurantifolia* oil, *Citrullus colocynthis* fruit, *Vernonia amygdalina* leaves, *Citrus paradise* fruit, *Alstonia boonei* stem bark, *Tetrapleura tetraptera* pods, and *Aloe vera* leaves, whereas *Zingiber officinale* rhizome was toxic at 2000 mg/kg. No toxicity report was found for *Momordica balsamina* and *Vigna subterranean*.

## 4. Discussion

### 4.1. Sociodemographic Information of the Informants Interviewed

A larger proportion of men constituted the study population as informants for traditional medicinal management of obesity. The reason may be that the majority of female herbalists are rather focused on maternal health care [595]. Most of the informants fell in the age bracket of 36–59 years. The age distribution was based on Nigeria's definition of youth being persons aged 18–35 years [596] and the elderly being 60 years and above [22]. Although it is generally assumed that elderly people are the major custodians of traditional knowledge [597], in this study, the majority of participants were recorded as being between 36 and 59 years of age. Most respondents had attained primary education and were rather herb sellers than herbal practitioners.

### 4.2. Distribution of Plant Species and Families

The family with the highest representation in terms of the number of species is Leguminosae (10 species), followed by Cucurbitaceae (8 species) out of a total of 39 families and 70 plant species used to traditionally manage obesity in the North Central States. A similar distribution of plant species among plant families has been reported for medicinal plants used for traditional maternal healthcare in Katsina State, Nigeria [595]. Among the 39 plant families, Euphorbiaceae, Myrtaceae, Piperaceae, and Rhamnaceae have been previously reported to contain plant species traditionally used to treat obesity [598]. Pare et al. [15] also documented plant species used in Burkina Faso to treat obesity. The findings on the plant families mentioned in the later study were similar to what is documented in the current study as well as in another study by Odukoya et al. [7] on medicinal plants used in treating cardiovascular diseases and associated risk factors, and the families include Apocynaceae, Arecaceae, Capparaceae, Combretaceae, Cucurbitaceae, Leguminosae, Malvaceae, Meliaceae, Moringaceae, Pedaliaceae, Poaceae, Rhamnaceae, Rubiaceae, and Rutaceae.

### 4.3. Method of Preparation and Plant Parts Used

The leaves of the plants were the most frequently used and decoction was the commonest method of preparation as was reported in other studies [599, 600]. In traditional medicine, leaves are widely used because they are relatively more abundant, available, and accessible in nature compared to other plant parts. The decoction method of preparation is widely used in traditional medicine for harnessing the beneficial effect of plants [601, 602]. Decoctions appear to be easier to ingest for the required number of times per day for a given length of treatment [603]. Furthermore, decoctions are commonly used as it is believed that boiling water extracts more active ingredients from the plants. Additionally, individuals who are conscious of their religion, mostly Muslims and a few Christians, prefer decoction as their religion does not permit them to consume alcohol. Powdered herbs were mostly mixed and/or dissolved in tea, *fura da nono* (cow milk), *kunu* (local drink made of sorghum), and *zobo* (local drink made of *H. sabdariffa*), i.e., as liquid foods.

### 4.4. Domestication Status and Life Forms of Medicinal Plants

There were more herbs than trees and shrubs used for obesity treatment/management, as well as more cultivated plants than those found in the wild. Since cultivated herbs are more in demand [604], there is little danger posed to the plants' biodiversity due to over usage and overexploitation as the plants could be cultivated in large farmlands. Herbaceous plants were found to have constituted the majority of traditional medicinal plants used in the treatment of obesity which is in line with other studies for various ailments [605, 606], although some have reported the use of shrubs than herbs [607, 608].

### 4.5. Dosage and Route of Administration

In this study, efforts were made to get specific information about the dosage of administration from the informants; however, the information provided concerning quantity administered during medication was limited and many times nonspecific. For example, reference was made to the quantity of prepared herb used as a cup (which meant a minimum of 250 mL), half a cup (≤ 100 mL), or a small cup (≤ 50 mL). When informants referred to the dosage as a small cup, they meant a standard small glass cup used for drinking hot drinks, which is gin in Nigeria. Although many informants specified the quantity in terms of the volume of decoction or juice used, there were gaps in the exact concentration. This is a serious concern for educated professionals who want to consume herbs as they are deterred because of the knowledge that some plants taken at a high dose may be harmful to their health [595]. The plants are consumed mainly through the oral route [609], and external use of the prepared plants' parts such as dermal application, body bath, soaking, or steaming methods was not reported. This corroborates with an earlier study which had shown that most plants used for weight reduction were mainly taken orally [15].

### 4.6. Percentage Values of Plants

Local inhabitants have used various plant species for managing or treating different ailments [610] and this study recorded the different species used in the traditional management of obesity. Some of the plant species which had a FC above 20 or were among the top plants used for traditional management of obesity based on their percentage values have been reported in the literature. For example, *Hibiscus sabdariffa* has been reported to be used for weight reduction purposes in South Brazil by herbalists [598].

Other plant species identified in this study and reported to be used in Burkina Faso to treat obesity include *Acacia nilotica, Adansonia digitata*, *Azadirachta indica*, *Ceratotheca sesamoides*, *Citrullus colocynthis*, *Citrus aurantifolia*, *Commiphora africana*, *Hibiscus sabdariffa*, *Khaya senegalensis*, and *Tamarindus indica* [15]. Approximately 15% of the plant species found in the North Central States in Nigeria are similar to those reported from Burkina Faso, which suggests similar traditions between the two West African Countries and could also indicate the need for sharing information to expand the knowledge horizon of traditional medicinal plants for obesity treatment in Africa.

Ethnobotanical uses of some of the medicinal plants recorded in this study for the purpose of obesity management have been previously reported. These plants/herbs include juiced *Allium cepa* with wine and honey [611]; infused *Allium sativum* seeds/clove [20]; juiced *Aloe barbadensis* leaves [612]; decoction of *Alstonia boonei* leaves [613]; decoction of *Carica papaya* leaves, fruit, seeds, and roots [613, 614]; decoction of *Citrus lanatus* fruit and roots [20, 613]; infused and juiced *Citrus aurantifolia* fruit [20, 613]; maceration and infusion of *Curcuma longa* rhizome [615]; *Psidium guajava* leaves [20]; decoction of *Vernonia amygdalina* leaves [613]; and decoction and juice of *Zingiber officinale* rhizome [613].

### 4.7. Antiobesity Pharmacological Effects of the Plants

Plants identified for their traditional medicinal uses against obesity with no report on their antiobesity pharmacological effects in literature were 23 plants including *Acacia nilotica, Boscia senegalensis, Chrozophora senegalensis, Commiphora africana, Detarium senegalensis, Guiera senegalensis, Heinsia crinita, Momordica balsamina*, and *Senna occidentalis*. This presents a scientific gap in the search for a cure for obesity from medicinal plants and there is a need to fill up this gap. Other plants such as *Adansonia digitata*, *Azadirachta indica*, *Brassica nigra*, and *Ocimum basilicum* have scanty reports about their pharmacological effects as related to obesity. These plants need to be well researched for their antiobesity pharmacological effects to discover antiobesity extracts/bioactive agents and/or give credence to their traditional medicinal uses.

Reports of the antiobesity activity for some of the documented plants were found in the literature. These plants with antiobesity effects might exert their potency in one or more of the following ways: inhibition of lipases activity or lipid absorption, suppression of food intake, inhibition of adipogenesis and lipogenesis, induction of apoptosis and lipolysis, stimulation of energy expenditure or thermogenesis, inhibition of inflammation, and modulation of hormones and endocrine functions [616].

Some of the plants that are well known for their inhibition of lipases/lipid absorption include *Allium cepa, Allium sativum, Citrullus colocynthis, Citrus aurantifolia, Citrus limon, Cocos nucifera, Curcuma longa, Hibiscus sabdariffa, Irvingia gabonensis, Momordica charantia, Persea americana, Psidium guajava, Spinacia oleracea, Tamarindus indica, Tetrapleura tetraptera*, and *Zingiber officinale*. Leaf, seed, and fruit extracts and fractions of *Capsicum annuum*, *Carica papaya*, *Brassica nigra*, *Citrullus colocynthis*, *Citrus limon*, *Momordica charantia*, *Mucuna flagellipes*, *Murraya koenigii*, *Ocimum gratissimum*, *Psidium guajava*, *Spinacia oleracea*, *Zingiber officinale,* and *Ziziphus jujube* were associated with in vitro inhibition and/or decreased activity of pancreatic lipase [42, 64, 100, 181, 182, 188, 206, 250]. In addition to the inhibition of PL, *Hibiscus sabdariffa* and *Tamarindus indica* also showed alpha-amylase inhibitory activity [42]. *Persea americana* inhibited acetyl-CoA carboxylase—which catalyzes the carboxylation of acetyl-CoA to create malonyl-CoA [190], *Vernonia amygdalina* decreased mRNA expression of FAS, LPL, and leptin, and *Piper nigrum* increased insulin and leptin sensitivity [196].

The inhibition of adipogenesis/lipogenesis and induction of lipolysis in fat cells known as the 3T3-L1 cells is a valid antiobesity approach for medicinal plants [617], and some of these plants include *Aloe vera, Capsicum annuum, Cinnamomum verum, Citrus aurantifolia, Citrus limon, Citrus paradise, Cocos nucifera, Cucurbita ficifolia, Ipomoea batatas, Irvingia gabonensis, Momordica charantia,* and *Persea americana*. In *Glycine max,* 7,3′,4′-trihydroxyisoflavone reduced lipid content and adipocyte differentiation [132]. In a similar manner, *Allium cepa*, *allium sativum*, *Curcuma longa*, *Piper nigrum*, *Psidium guajava*, *Zingiber officinale*, and *Hibiscus sabdariffa* were found to have inhibited lipogenesis in 3T3-L1 adipocytes and enhanced lipolysis while preventing lipid accumulation [26, 34, 120, 146, 198, 200, 244].

Sung, Bang, and Lee [72] reported that caposicosides A and G from *Capsicum annuum* reduced intracellular lipid buildup in these cells. *Cucurbita ficifolia* chloroform extract was found to have attenuated adipogenesis in human mesenchymal stem cells [116]. *Curcuma longa*, cinnamaldehyde from *Cinnamonum verum,* and polyphenolic extract of *Hibiscus sabdariffa* likewise prevented adipocyte differentiation and adipogenesis in the 3T3-L1 cells [34, 120, 145]. Also, leaf extracts of *Brassica oleracea*, *Moringa oleifera,* and *Ocimum basilicum* inhibited adipogenesis in 3T3-L1 adipocytes [63].

Some pathways have also been reported through which some of these plants carry out their pharmacological activities. The coordinated gene expression of multiple adipogenic genes, including FAS and LPL, is what regulates adipogenesis [618] which goes on to imply that the dietary or natural compounds that suppress either of them and the adipogenic process in turn will significantly affect the prevention and treatment of obesity. This coordinated gene expression is regulated by a variety of transcription factors such as PPAR, AMPK, and C/EBP [617]. Fruit extract and chloroform fraction of *Ziziphus jujube* inhibited adipogenesis by decreasing the expression of PPARγ, C/EBPα, and β in 3T3-L1 preadipocytes [253]. High hydrostatic pressure extract of ginger increased fecal lipid extraction through the regulation of microRNA-21/132 expression and AMPK activation in white adipose tissue of rats fed an HFD [234].

The following plants are reported to stimulate energy expenditure or thermogenesis: *Allium cepa, Allium sativum, Aloe vera, Citrus paradise, Glycine max, Momordica charantia, Moringa oleifera,* and *Zingiber officinale*. Capsaicin from *Capsicum annuum* induced thermogenesis in mice fed with HFD and reduced insulin resistance and hepatic steatosis by PPARγ and TRPV-1 expression/activation; it also modulated adipokine gene expression in adipose tissues from obese mice and upregulated the expression of UCP2, PPARγ, and PPARα while downregulating the expression of leptin [73, 79].

The modulation of obesity-related hormones and endocrine functions was partly demonstrated by *Tamarindus indica, Tetrapleura tetraptera, Vernonia amygdalina, and Zingiber officinale* to mention a few [212, 218, 221]. Decrease in mRNA levels of adipose leptin and resistin resulted in mice on HFD with methanol seed extracts of *Momordica charantia* [170]. *Cinnamomum verum* altered ghrelin in obese mice [90]. Soybean protein isolates reduced weight gain and adipose tissue mass and increased GLP-1 secretion in mice as also did *Hibiscus sabdariffa* [133, 151]. In addition, *Citrullus colocynthis, Cinnamomum verum, Piper nigrum, Vernonia amygdalina,* and *Zingiber officinale* were reported to suppress food intake [221, 229]. For instance, flavonoid-rich extract from *Spinacia oleracea* had appetite-suppressing effects [205].

A few studies have documented how some of these plants have effected weight loss in humans as well; in humans with BMI ≥ 27, aqueous flower extract of *Hibiscus sabdariffa* was found to have reduced obesity and abdominal fat, while attenuating liver steatosis [144]. Capsaicin from *Capsicum annuum* increased GLP-1 and satiety while it decreased ghrelin, energy, and fat intake in healthy persons and was additionally found to have increased diet-induced thermogenesis in Japanese women [77, 82]. *Cocos nucifera* reduced abdominal obesity in women as well [109].

### 4.8. Toxicity Profile of Plants

According to the Organization for Economic Cooperation and Development (OECD) guidelines for testing chemicals, plant extracts and fractions with LD_50_ values > 2000 mg/kg body weight in rodents (acute toxicity testing) are classified as having low toxicity [619]. There were 61 plants that had LD_50_ values > 2000 mg/kg when administered either orally or intraperitoneally to rats and/or mice. The plants with LD_50_ values < 2000 mg/kg are thus considered potentially toxic and careful consideration should be applied to their use [620]. For example, oral acute toxicity studies revealed *Citrullus colocynthis* was toxic in rats, while *Garcinia kola* and *Guiera senegalensis* were toxic in mice (Table 4).

Subacute toxicity studies on plants usually involve the administration of repeated doses of the extracts/fractions for a period of 28 days in animals [466]. Plants that were reported as relatively more toxic in rodents in subacute toxicity studies at doses ≤ 300 mg/kg are *Cucurbita pepo* stem, *Citrullus colocynthis* seeds, *Azadirachta indica* bark, and *Vernonia amygdalina* leaves. Subchronic and chronic toxicity studies revealed more plants that were toxic to rodents at concentrations less or equal to 500 mg/kg and include *Citrullus colocynthis* fruit, *Murraya koenigii* leaves, *Citrus aurantifolia* oil, *Vernonia amygdalina* leaves, *Citrus paradise* fruit, *Alstonia boonei* stem bark, *Tetrapleura tetraptera* pods, and *Aloe vera* leaves. As expected, these studies provided information on the major toxic effects of the plants in rodents and the extent of organ damage. No toxicity report was found for *Momordica balsamina* and *Vigna subterranean*. This presents a scientific gap that requires to be filled by researchers interested in the toxicity of plants.

The assumption that medicinal plants are nontoxic because they are found or cultivated naturally in the environment, used medicinally for thousands of years, or are sometimes consumed as food could be misleading [621]. In this study, the toxicity potential of some of the plants used traditionally in the management of obesity reiterates the growing concern about the safety of traditional herbal medicines [622, 623]. Several factors which contribute to toxicity in the traditional use of medicinal plants include plant bioactive constituents, herb–drug interactions, high dosage, poor and inconsistent manufacturing practices, adulteration, and poor regulatory measures [624, 625]. Some traditional remedies are a combination of two or more plants or substances, and as much as such combinations may increase the desired effect, there are possibilities of occurrence of or increased adverse reactions to the consumer [609]. These potential adverse effects emphasize the importance of toxicity testing and the safety assessment of traditional remedies for human consumption. The reasons people take decoctions of potentially toxic medicinal plants to treat obesity may be attributable to ignorance of its toxic effects or the perception of safety when taking the dose prescribed by the herbal practitioner [624].

### 4.9. Limitations of the Study

Limitations of the study include the language barrier that prevented us from gleaning much-desired information from some of the herbal practitioners that seemed to have more to say than was obtained and reported despite the use of interpreters. In some cases, some of them refused to freely supply information on some of the plants as they wanted to be paid handsomely while others claimed it was their family or ancestorial secrets. The study was quite expensive as some herbal practitioners had to be paid to source the different parts of the plants for proper identification. Finally, some herbal practitioners were not certain about the dosage as the prescription from person to person varies depending on age, gender, body size, and tolerance.

## 5. Conclusions

This is the first study that provides a full inventory of medicinal plants traditionally used in the treatment of obesity in the given states in Nigeria. Although scientific evaluation of the pharmacological activities pertaining to obesity and toxicological activities of some of the surveyed plants exists, research is needed for plants that have not been studied to validate the traditional claims. Further studies on the mechanisms of action of plants with antiobesity potentials are warranted, especially where no reports were obtained. Further toxicity studies are required to ensure the safety of use (both acute and chronic) of plants using preclinical and clinical models. Also, the plants which were most cited could further be pharmacologically evaluated and developed into herbal products that are cheap and accessible to the populace.

## Figures and Tables

**Table 1:** Demographic characteristics of interviewed informants (*N* = 700).

Participant data	Number	%
Gender		
Male	463	66.1
Female	237	33.9
Age (yr)		
18–35	102	14.6
36–59	406	58.0
> 60	192	27.4
Educational level		
Primary	329	47.0
Secondary	151	21.6
Tertiary	8	1.1
None	212	30.3
Participant trade		
Herbal practitioners	267	38.1
Herb sellers	433	61.9

**Table 2:** Plants used to treat or manage obesity in the North Central States.

S/N	Botanical name	Local name/language	Common name	Family	Voucher specimen number	Plant type	C, W, W&C	Parts used	MoP	Preparation	Route of administration/dosage	FC	RFC	% value
1	*Acacia nilotica* (L.) Delile	Bagaruwa (H)	Gum arabic tree; Scorpion mimosa	Leguminosae	B03709	T	W	S, F	D	Ground into a powder	Oral; add 2 teaspoons to locally made boiled drink	8	0.0114	0.81
2	*Adansonia digitata* L.	Kuka (H)	Baobab	Malvaceae	M02512	T	W	L	D, M	Macerated in ethanol or boiled in water	Oral; drink a small cup daily	9	0.0129	0.92
3	*Allium cepa* L.	Albasa (H)	Onions	Amaryllidaceae	GA135-3621	H	C	L, B	D, M	Macerated in ethanol or boiled in water	Oral; drink a small cup daily	10	0.0143	1.02
4	*Allium sativum* L.	Tafarnuwa (H)	Garlic	Amaryllidaceae	GA135-3622	H	C	C	D, M	(1) Add powdered garlic to a meal daily; (2) mash cloves, and add to a cup of water or juice; (3) eaten raw	(1) A tea spoon added in preparing a meal; (2) orally, drink a cup daily; (3) one or two large garlic cloves per meal	29	0.0414	2.95
5	*Aloe vera* (L.) Burm.f., *Aloe barbadensis* Mill	Aloe vera (H), Ahon erin (Y)	Aloe vera	Xanthorrhoeaceae	UAM/FH/314	H	C	L	J	(1) Cut open the leaf and scoop the gel with a teaspoon; (2) grind with the skin to extract juice, which is sieved	(1) Add a teaspoon of gel to a cup of juice and drink; (2) drink a small cup in the morning and night before sleeping	25	0.0357	2.55
6	*Alstonia boonei* De wild.	Ahun (Y), Egbun (I)	Cheese wood	Apocynaceae	GA133-2752	T	W	Sb	M	Cut the stem bark into pieces and soak it in alcohol	Drink ¼ cup in the morning and evening	16	0.0229	1.63
7	*Ananas comosus* (L.) Merr.	Abarba (H)	Pineapple	Bromeliaceae	B03560	H	C	F	M	Shred bark and blend to obtain a juice	Drink a cup of juice twice daily	9	0.0129	0.92
8	*Anthocleista vogelii* Planch.	Kwari (H), Sapo (Y), Mpoto (I)	Cabbage tree	Gentianaceae	GA134-7421	T	W	R	M	Cut the root bark and place it in a bottle of alcohol	Drink ¼ cup daily	17	0.0243	1.73
9	*Azadirachta indica* A.Juss.	Dogon yaro (H)	Neem tree	Meliaceae	UAM/FH/0315	T	WC	L	D	Crushed leaves are boiled in water	Oral; a cup daily	15	0.0214	1.53
10	*Beta vulgaris* L.		Beetroot	Maranthaceae	B03510	H	C	R	D	Boil in hot water to extract content	Oral; a cup daily	10	0.0143	1.02
11	*Boscia senegalensis* Lam.	Anza, hanza (H)	Arabic	Capparaceae	B03520	S	W	St, Br	D	Boil in hot water to extract content	Oral; a cup daily	11	0.0157	1.12
12	*Brassica oleracea* L.	Kabeji (H)	Cabbage	Brassicaceae	B03530	H	C	L	D	(1) Slice and eat fresh; (2) add leaves to onions	Oral; (1) eat ½ plate; (2) drink ½ cup 3 times a day	7	0.0100	0.71
13	*Brassica nigra* (L.) K.Koch	Kõmayya (H)	Black mustard seed	Brassicaceae	B03531	H	C	S	P	Grind into a powder and add to a meal	Add a teaspoon to each meal and eat 3 times daily	8	0.0114	0.81
14	*Capsicum annuum* L.	Shombo/Shomo Dogo dogo (H)	Chili pepper, cayenne pepper	Solanaceae	B03540	H	C	F	P, D	(1) Dried and ground/used as harvested; (2) grind and add to food	(1) Use 2 teaspoons as a spice in food preparation and eat; (2) add ½ teaspoon to a tea and drink twice daily	11	0.0157	1.12
15	*Capsicum chinense* Jacq.	Atarigu (H)	Adjuma, Ají Dulce	Solanaceae	B03541	H	C	F	J	Grind/blend pepper and add to lemon juice	Drink ½ cup daily of juice	9	0.0129	0.92
16	*Carica papaya* L.	Ibepe (Y)	Pawpaw	Caricaceae	UAM/FH/0311	T	C	F	J	Blend and drink the juice	Oral; a cup daily of juice	14	0.0200	1.43
17	*Ceratotheca sesamoides* Endl.		False sesame	Pedaliaceae	B03601	H	C	S, L	D	Boil in hot water to extract content	Oral; a cup daily of decoction	7	0.0100	0.71
18	*Chrozophora senegalensis* (Lam.) A.Juss. ex Spreng.	Mingirya, dàmágì (H)		Euphorbiaceae	B03611	S	W	Sb	P, D	Stem bark is ground into a fine powder and mixed with local drinks such as pap/*kunu*	½ tablespoon of powder is added to a locally made drink, taken twice a day	11	0.0157	1.12
19	*Cinnamomum verum* J.Presl	Kirfa [H], Ohio (I)	Cinnamon	Lauraceae	B03620	T	WC	F	P, D, M	Dried and ground into a fine powder	Used as a spice in food (a teaspoonful), added to tea or locally prepared drinks such as pap and kunu	23	0.0329	2.34
20	*Citrullus colocynthis* (L.) Schrad.	Bara (Y), Guna (H), egusi elili (I)	Bitter lemon, bitter apple, egusi melon, wild gourd	Cucurbitaceae	UAM/FH/0312	H	C	W	D	(1) Cooked or soaked. Put into a pot without water and cooked till soft, and extract the water. May be mixed with Bama mayonnaise. (2) Peel and pound it, then add water. May be mixed with Maltina or lime	Drink a cup of water extract once a day	9	0.0129	0.92
21	*Citrullus lanatus* (Thunb.) Matsum. & Nakai	Kankana (H)	Watermelon	Cucurbitaceae	B03630	H	C	S	J	Blend dried seeds and add to juice or use seeds as tea	Oral; a cup daily of juice	12	0.0171	1.22
22	*Citrus aurantifolia* (Christm.) Swingle	Lemu tsami (H)	Lime	Rutaceae	B03640	T	WC	F	J	Wash the fruit, slice, squeeze out the juice, and drink	Oral; a cup daily of juice	35	0.0500	3.56
23	*Citrus limon* (L.) Burm. f.	Baban lemun tsami (H), Oroma nkirisi (I)	Lemon	Rutaceae	B03641	T	C	L, St, R, F, Fw	D, J, M	(1) Boil stem/root; (2) wash fruit, slice, squeeze out the juice, and drink	(1) Juice: drink a cup daily; (2) fruits: eat no more than 3 a day	32	0.0457	3.26
24	*Citrus paradise* Macfad.	Garehul (H)	Grapefruit	Rutaceae	B03642	T	C	F	J	Taken as harvested/blended to form a juice	(1) Juice: drink a cup daily; (2) fruits: eat not more than two per day	16	0.0229	1.63
25	*Cochlospermum tinctorium* Perrier ex A.Rich.	Rawaya, Kyambas (H)		Bixaceae	B03651	H	W	St, R	D, M	Boil in hot water to extract content; soak in alcohol	A cup daily if in water, if in alcohol ¼ cup daily	10	0.0143	1.02
26	*Cocos nucifera* L.	Kwakwa (H)	Coconut	Arecaceae	UAM/FH/0316	T	WC	F	J	Remove the white part of the fruit, blend and filter to obtain the milk. Leave the milk for 24 h to separate the oil from the milk	Use oil for cooking meals daily	18	0.0257	1.83
27	*Commiphora africana* (A.Rich.) Endl.	Wurishi (H)	African silk tree	Burseraceae	B03660	T	W	Sb	D, M	Stem bark is ground into a fine powder and soaked in water	Drink a small cup 3 times daily before every meal	16	0.0229	1.63
28	*Cucumis sativus* L.	Kukumba (I), Kokwamba (H)	Cucumber	Cucurbitaceae	B03631	H	C	F	J	Blend the whole cucumber and drain out the juice	Drink 1 cup in the morning and at night	21	0.0300	2.14
29	*Cucurbita ficifolia* Bouché		Pumpkin	Cucurbitaceae	B03632	H	C	L	D	Boil in hot water to extract content	Oral; a cup daily of decoction	9	0.0129	0.92
30	*Cucurbita pepo styriaca* L.	Ogu (I)	Pumpkin	Cucurbitaceae	B03633	H	C	S	J, D	Dry the seeds, grind and add to water to make them into juice	Oral; a cup daily of juice	14	0.0200	1.43
31	*Curcuma longa* L.	Gangamau (H), Kurkur (H)	Turmeric	Zingiberaceae	B03670	H	C	R	P, D	Grind to a powder, add to locally made drink (either *kunu* or pap), or used as a spice in food	A teaspoon is added to a cup of hot water and taken 2 times daily	12	0.0171	1.22
32	*Cymbopogon citratus* (DC.) Stapf	Lemun tsami ciyawa (H), Toho-gile (T)	Lemon grass	Poaceae	UAM/FH/0310	H	C	L	J, D	Wash, blend/grind and filter and drink the juice	Oral; a cup daily of juice	14	0.0200	1.43
33	*Cyperus esculentus* L.	Aya (H)	Tiger nut, yellow nutsedge	Cyperaceae	B03690	H	C	F	D	Make a local drink of tiger nut such as kunu by grinding	Drink three to four cups a day	15	0.0214	1.53
34	*Detarium senegalensis* J.F.Gmel.	Runhu (H)	Sweet datar, wild cassia	Leguminosae	B03700	T	WC	S	P	Ground to a powder	Mix with locally produced drinks such as kunu, pap, or even tea and honey; may be used as a spice in food	9	0.0129	0.92
35	*Fragaria vesca* L.	Garin bambaro (H)	Strawberry	Rosaceae	B03711	H	C	F	J	Fruits could be eaten raw or made into a juice	A cup of juice taken orally 3 times a day	27	0.0386	2.75
36	*Gongronema latifolium* Benth.	Utazi (I)		Apocynaceae	GA133-2754	H	C	L, S	D	Boil in hot water to extract content; soak in alcohol	Hot water: drink a cup daily; alcohol: drink ¼ cup daily	21	0.0300	2.14
37	*Garcinia kola* Heckel.	Cida goro (H) Orogbo (Y)	Bitter kola	Clusiaceae	B03720	H	WC	F	M	Peel off the bark; slice seeds into pieces and soak in alcohol	Chew 2 seeds daily or drink a small cup once daily	30	0.0429	3.05
38	*Glycine max* (L.) Merr.	Waken soya (H)	Soybeans	Leguminosae	B03701	H	C	S	D	Heat beans in a pan, grind, filter and cover the filtered milk for 24 h. Separate the oil for use	Add a teaspoon of oil to each meal daily	11	0.0157	1.12
39	*Gnetum africanum* Welw.	Okazi (I), Afang (Efik), àjáàbalè (Y)		Gnetaceae	B03740	H	C	L	D, M	Boil in hot water to extract content; soak in alcohol	Hot water extract: drink a cup daily; Alcohol extract: drink ¼ cup daily	12	0.0171	1.22
40	*Guiera senegalensis* J.F.Gmel.	Sabara (H)	Moshi medicine	Combretaceae	B03571	S	C	L, St	D	Ground into a fine powdered paste	½ tablespoon added to a locally made drink or a cup of Lipton tea	8	0.0114	0.81
41	*Heinsia crinita* (Afzel.) G.Taylor		Bush apple	Rubiaceae	B03731	S	C	L	D	Boil in hot water to extract content	Oral; a cup daily of decoction	7	0.0100	0.71
42	*Hibiscus sabdariffa* L.	Zobo (H)	Roselle	Malvaceae	UAM/FH/0308	H	C	L, F	D	Boil leaves with 2 cups of water, cool, and strain	Oral; two cup daily of decoction	22	0.0314	2.24
43	*Ipomoea batatas* (L.) Poir.	Dankali (H)	Sweet potato	Convolvulaceae	UAM/FH/0321	H	C	R	D	Cook and eat; grind when dried and add to juice	Eat a plate or add 2 teaspoons of powder to a cup of juice	12	0.0171	1.22
44	*Irvingia gabonensis* (Aubry-Lecomte ex O'Rorke) Baill.	Ogbono (I)	Wild mango	Irvingiaceae	A06926	T	WC	S	D, M	Grind dry seeds, and use them to prepare a soup	Enough for a meal	10	0.0143	1.02
45	*Khaya senegalensis* (Desv.) A.Juss.	Haa (T)	African mahogany	Meliaceae	UAM/FH/0322	T	WC	Sb	D	Boil or soak in water	Oral; drink a cup in the morning and evening	7	0.0100	0.71
46	*Luffa aegyptiaca* Mill.	Sponge soap	Luffa	Cucurbitaceae	B03634	H	WC	L	J, D	Boil in water or soak in alcohol	Oral; drink a cup in the morning and evening	19	0.0271	1.93
47	*Lycopersicon esculentum* Mill.	Tomatur (H)	Tomato	Solanaceae	UAM/FH/0307	H	WC	F	J	Wash fruit, blend to a juice	Oral; a cup daily of decoction	7	0.0100	0.71
48	*Mentha pulegium* L.	Na'a na'a (H)	Mint leaf	Lamiaceae	B03760	H	C	L	D	Boil in hot water to extract content	Oral; a cup daily of decoction	10	0.0143	1.02
49	*Momordica balsamina* L.	Garaguni, Garahuni (H)	Balsam apple, African cucumber	Cucurbitaceae	B03635	H	WC	L	D	Grind leaves, mix with a cup of tea	Oral; a cup daily of decoction	10	0.0143	1.02
50	*Momordica charantia* L.	Daddegu (H)	Bitter melon	Cucurbitaceae	B03636	H	WC	F	J, D	Fruit eaten raw, or made into a juice and mixed with other fruits/honey to reduce the bitter taste	Oral; a cup daily of juice	8	0.0114	0.81
51	*Moringa oleifera* Lam.	Zogole (H)	Moringa, drumstick tree, miracle tree	Moringaceae	UAM/FH/0313	H	C	L, St, S, R	D, P, M	Boil leaves in water and drink; leaves may be included in food; grounded seeds, stems and roots may be added to local drinks; seeds chewed in raw form	Drink ½ cup; eat enough leaves for a meal	15	0.0214	1.53
52	*Mucuna flagellipes* Hook.f.		Ukpo	Leguminosae	B03702	T	C	S	D, M	Boil in hot water to extract content; soak in alcohol	Hot water extract: a cup daily; alcohol extract: ¼ cup daily	18	0.0257	1.83
53	*Murraya koenigii* (L.) Spreng.	Curry	Curry leaf	Rutaceae	B03643	T	C	L	D	Eaten fresh or half cooked	Eat ½ a plate once daily	14	0.0200	1.43
54	*Myristica fragrans* Houtt.	Gyadar Kamshi (H)	Nutmeg	Myristicaceae	B03770	T	WC	F, S	P	Ground till fine powder	A teaspoon is added as a spice to food daily	9	0.0129	0.92
55	*Ocimum basilicum* L.	Efirin (Y)	Basil	Lamiaceae	B03761	H	C	L	J	Wash, pound, or grind to obtain juice	Oral; a cup of juice daily	9	0.0129	0.92
56	*Ocimum gratissimum* L.	Doddoya (H)	Scent leaf, fever plant, fever bush	Lamiaceae	B03762	H	C	L	D	Wash, add to the meal, and eat; boil in hot water to extract content	Eat leaf in a plate of food or drink half cup daily	20	0.0286	2.04
57	*Persea americana* Mill.	Piya (H)	Avocado pear	Lauraceae	B03621	T	C	L, F	J	The succulent part without the skin and seed is eaten	At least 1 fruit twice per day	8	0.0114	0.81
58	*Piper guineense* Schumach. & Thonn.	Uziza (I)	Uziza seeds, Ashanti pepper, Benin pepper	Piperaceae	B03780	H	C	L	D	Cut leaves or ground seeds are used in making a local watery dish called ‘pepper soup'	Drink a bowl (of 300 mL) of the pepper soup	11	0.0157	1.12
59	*Piper nigrum* L.	Barkono (H), Yiye (Y)	Black pepper	Piperaceae	B03781	H	C	F	D, J	(1) Dried, ground, and used as a spice in food; (2) grind to obtain a juice, add honey, and drink	A teaspoon daily in food is eaten	13	0.0186	1.32
60	*Prosopis africana* (Guill. & Perr.) Taub.	Kirya (H)	False locust	Leguminosae	UAM/FH/0309	T	WC	Sb	D, M	Stem bark is dried, ground to a powder, and mixed with other herbs in water or alcohol	Drink a small cup 3 times a day	8	0.0114	0.81
61	*Psidium guajava* L.	Ganyen gwava (H)	Guava leaves	Myrtaceae	UAM/FH/0320	T	WC	L	D	Washed and cooked to obtain a tea	Oral; ½ cup 3 times a day before/after meals	10	0.0143	1.02
62	*Pterocarpus mildbraedii* Harms		Ora, Oha	Leguminosae	B03704	T	W	L	D, M	Boil in hot water to extract content; soak in alcohol	Hot water extract: drink a cup once daily; Alcohol extract: drink ¼ cup daily	26	0.0371	2.65
63	*Senna occidentalis* (L.) Link	Rai daure/Râi ɗòòré (H)	Coffee senna	Leguminosae	B03705	H	C	L	D	Leaves are ground and mixed with a cup of tea	Oral; a cup daily	8	0.0114	0.81
64	*Spinacia oleracea* L.	Alewo, Alayyahu (H)	Spinach	Amaranthaceae	B03800	H	C	L	D, J	(1) Wash and cut leaves and add to a meal or eat half cooked; (2) make spinach juice to drink	Eat a plate per meal; drink ½ cup daily of juice	8	0.0114	0.81
65	*Tamarindus indica* L.	Tsamiya (H)	Tamarind	Leguminosae	B03706	T	C	S, F	D	(1) Boil fruits and use in local drinks like *kunu zaki* and *kunu geda*; (2) Boil in water for ∼5 min, allow to cool before mashing with your hands to bring out the sour taste, then sieve and store	(1) Chew or leak 5–7 pod-like fruits twice daily, (2) Drink a cup with a teaspoon of honey 2 times daily	10	0.0143	1.02
66	*Tetrapleura tetraptera* (Schumach. & Thonn.) Taub.		Aiden pod	Leguminosae	B03707	T	W	P	D	Cut leaves or ground seeds are used to make a local watery dish called ‘pepper soup'	Drink a bowl (300 mL) of pepper soup once daily	16	0.0229	1.63
67	*Vernonia amygdalina* Delile	Tyuna (T), Shuwaka (H), Ewuro (Y), Onugbu (I)	Bitter leaf	Compositae	B03820	S	C	L	J, D, M	Wash, squeeze, sieve, and drink; boil in water or soak in alcohol	Drink a cup of juice extract once daily; hot water extract: drink a cup twice a day; Alcohol extract: drink ½ cup daily	21	0.0300	2.14
68	*Vigna subterranea* (L.) Verdc.	Gujiya (H)	Bambara groundnut	Leguminosae	B03708	H	C	S	D	Cook nuts or grind nuts and cook them as part of a meal	Eat a plate of coked Bambara nut for a meal	9	0.0129	0.92
69	*Zingiber officinale* roscoe	Chetah/Cìttáá mài ƙwààyáá (H), Jinja (I), Ata-ile (Y)	Ginger	Zingiberaceae	UAM/FH/0317	H	C	R	D, J, M	(1) Ground into a powder and used in food or mixed with water; (2) boil ground ginger with water, sieve to separate the chaff from the juice, and then drink; (3) scrape or peel off the back, then grind into juice or soak in alcohol	(1) One tablespoon for food preparation and more for drinks; (2) ½ cup daily; (3) chew fresh ginger roots	30	0.0429	3.05
70	*Ziziphus jujube* Mill.	Magarya (H)	Jujube fruit	Rhamnaceae	B03830	S	WC	L	P	Grind leaves and add a spoon to a cup of milk tea	Oral; add a teaspoon of powdered leaves to tea and drink a cup daily	7	0.0100	0.71

*Note:* Grown state: C: cultivated; W: wild; C&W: cultivated/wild. H: Hausa, Y: Yoruba, I: Ibo, and T: Tiv. Parts used: B: bulb, Br: branch, C: cloves, F: fruits, Fw: flower, L: leaves, P: pod, R: roots, S: seeds, Sb: stem bark, St: stem, and W: whole. Plant type: H: herb, T: tree, and S: shrub. MoP: method of preparation; D: decoction, J: juice, M: maceration, and P: powder. Dosage: a cup (≥ 250 mL), half a cup (≤ 100 mL), or a small cup (≤ 50 mL). % value: Fc/*N*, where *N* is the total number of respondents and Fc is the number of respondents who cited a particular plant species. FC: frequency of citation (the number of times the plant was mentioned by respondents).

**Table 3:** Plants reported as being used to treat or manage obesity and their pharmacological activity.

S/N	Botanical name	Common name	Antiobesity effect of plant parts/types observed in in vitro or in vivo studies
1	*Acacia nilotica* (L.) Delile	Gum arabic tree; Scorpion mimosa	None reported

2	*Adansonia digitata* L.	Baobab	• Fruit increased satiety in humans [25]

3	*Allium cepa* L.	Onions	• Ethyl acetate extract of bulb inhibited fatty acid synthase in cancer cells and 3T3-L1 adipocytes [26]
			• Bulb oil decreased body mass and adipose tissue in rats fed HFD [27]
			• N-acetylcysteine isolated from the bulb extract reduced body weight and BMI in HFD obese rats [28]
			• Peel extract inhibited pancreatic lipase in vitro and reduced body and adipose tissue weights in mice fed HFD [29]
			• Quercetin-rich peel extract inhibited the differentiation of 3T3-L1 preadipocytes and adipogenesis in rats fed HFD [30–32]
			• Aqueous bulb extracts reduced body weight and fat weight and suppressed differentiation of white preadipocyte cells in Zucker diabetic fatty rats [33]
			• Bulb extract displayed antiadipogenic effects in preadipocyte 3T3-L1 cells [34]
			• Leaf and bulb juice extracts inhibited pancreatic lipase activity in vitro [35, 36]

4	*Allium sativum* L.	Garlic	• S-Methyl L-cysteine, S-allyl-l-cysteine sulphoxide, and S-allyl cysteine were shown to have antiobesity activities via regulating lipid metabolism in diet-induced obesity in rats [37, 38]
			• Oil decreased body weight and adipose tissue mass in rats fed HFD [27]; prevented obesity-triggered NAFLD in mice fed HFD [39]
			• Garlic supplementation in feed reduced body weight, white adipose tissue mass, and mRNA levels of adipogenic genes and enhanced thermogenesis in mice fed HFD [40]; decreased body weight and epididymal fat accumulation, ameliorated liver steatosis, and downregulated the expression patterns of epididymal adipose tissue genes [41]
			• Methanol bulb extract displayed pancreatic lipase inhibitory activity in vitro [42]
			• Stem extract decreased body weight gain and WAT cell size and restored adiponectin and leptin to near normal levels in obese mice fed an HFD [43]
			• The extract obtained through high hydrostatic pressure decreased weight gain in rats fed a HFD [44]
			• Aged black garlic decreased body and adipose tissue masses in rats fed a HFD [45]
			• Ajoene inhibited adipogenesis and induced apoptosis in 3T3-L1 cells [46]
			• Bulb extract displayed antiadipogenic effect in preadipocyte 3T3-L1 cells [47, 48]

5	*Aloe barbadensis* Mill Synonym *Aloe vera* (L.) Burm.f.	Aloe vera	• Gel reduced body fat accumulation via modulation of energy expenditure in DIO rats [49]; inhibited adipose LPL and activated HSL in obese rats [50]
			• Aloe gel complex reduced insulin resistance, body weight, and fat mass in obese people [51, 52]
			• Lophenol and cycloartanol reduced visceral fat mass [53]; increased the PPAR expression levels in obese mice [54]

6	*Alstonia boonei* De Wild.	Cheese wood	• Ethanol extracts of the stem bark diminished body weight and fat accumulation in obese rats [55, 56]

7	*Ananas comosus* (L.) Merr.	Pineapple	• Fruit juice reduced body weight [57, 58]; reduced fat accumulation; and regulated the expression of obesity-related genes in rats [59]

8	*Anthocleista vogelii* Planch.	Cabbage tree	• Ethanol and chloroform extracts of the root bark diminished body weight and fat mass in obese rats and obese diabetic rats, respectively [60, 61]

9	*Azadirachta indica* A.Juss.	Neem tree	• Aqueous and methanol extracts inhibited pancreatic lipase in vitro [62]

10	*Beta vulgaris* L.	Beetroot	None reported

11	*Boscia senegalensis* Lam.	Arabic	None reported

12	*Brassica oleracea* L.	Cabbage	• Leaf extract inhibited pancreatic lipase and adipogenesis activities in 3T3-L1 cells [63]

13	*Brassica nigra* (L.) K.Koch	Black mustard seed	• Leaf extract inhibited pancreatic lipase in vitro [64]

14	*Capsicum annuum* L.	Chili pepper, cayenne pepper	• Methanol extract and 9-oxooctadeca-10,12-dienoic acids inhibited acetyl-coenzyme A carboxylase in vitro [65]
			• Capsinoids suppressed body fat accumulation in mice [66] and diet-induced obesity via UCP-1-dependent mechanism [67]
			• Capsaicin modulated adipokine gene expression in adipose tissues from obese mice [68]
			• Capsicoside G-rich fraction from seeds lowered body weight and fat mass and inhibited adipogenesis in obese mice [69, 70]
			• Defatted ethanol extract of the seed decreased body weight and epididymal fat in HFD-induced obesity in C57BL/6J mice [71]
			• Capsicosides A and G isolated from seed extracts reduced intracellular lipid buildup in 3T3-L1 cells [72]
			• Fruits upregulated the expression of UCP2, PPARγ, PPARα, and adiponectin, and the expression of leptin was downregulated in obese rats [73]
			• Flowers inhibited pancreatic lipase in vitro [74]
			• Methanol seed extract displayed antiadipogenic activities by reducing the expression of PPARγ, C/EBPβ, and C/EBPα in 3T3-L1 fat cells [75]
			• Capsaicin-induced thermogenesis in mice fed an HFD [76]
			• Capsaicin increased the level of GLP-1 and satiety and decreased ghrelin, energy, and fat intake in healthy persons [77]
			• Methanol fruit extract inhibited adipogenic activity in 3T3 L1 fat cells [78]
			• Capsaicin reduced insulin resistance and hepatic steatosis by PPARα and TRPV-1 expression/activation in obese mice fed an HFD [79]; inhibited adipocyte differentiation process by the activation of AMPK in 3T3-L1 fat cells [80]
			• Water extracts from the fruit decreased activity of lipoprotein lipase in 3T3-L1 fat cells [81]
			• Red pepper elevated diet-induced thermogenesis in Japanese women fed an HFD and HCD [82]

15	*Capsicum chinense* Jacq.	Adjuma, Ají Dulce	None reported

16	*Carica papaya* L.	Pawpaw	• Leaf extracts inhibited pancreatic lipase in vitro [83]
			• Water extracts of the seed inhibited pancreatic lipase activity in vitro [84]
			• Fruit extract decreased body weight and hepatic triglycerides in HFD-fed rats [85]
			• Root extracts diminished fat mass and body weight in obese mice [86]

17	*Ceratotheca sesamoides* Endl.	False sesame	None reported

18	*Chrozophora senegalensis* (Lam.) A.Juss. ex Spreng.		None reported

19	*Cinnamomum verum* J.Presl	Cinnamon	• Water extracts decreased lipid and glycogen buildup in obese rat liver [87]
			• Aqueous bark extracts diminished body weight, fat mass, and leptin in obese diabetic rats [88]
			• Cinnamaldehyde prevented adipocyte differentiation and adipogenesis in 3T3-L1 fat cells [89], decreased body weight, suppressed appetite, and altered ghrelin in obese mice [90]
			• Methanol extracts prevented adipocyte differentiation and adipogenesis in 3T3-L1 preadipocytes [91]
			• Cinnamon extract increased insulin sensitivity and lowered fat in the brain and liver of obese mice, respectively [92]

20	*Citrullus colocynthis* (L.) Schrad.	Bitter lemon, bitter apple, egusi melon, wild gourd	• Alkaloids and glycosides extracted from the seeds decreased body mass and modulated hormones and metabolites in Wistar rats fed an HFD [93]
			• Fruit extract diminished body weight and food consumption in HFD-fed mice [94] and inhibited in vitro pancreatic lipase activity [95]
			• Oil from fruits triggered body weight reduction in mice [96]
			• Flesh extract suppressed adipogenesis in 3T3-L1 preadipocytes [97]; reduced abdominal fat index and hepatic lipids in HFD-fed mice [98]

21	*Citrullus lanatus* (Thunb.) Matsum. & Nakai	Watermelon	• Methanol leaf and seed extracts were found to possess pancreatic lipase activity [99, 100]

22	*Citrus aurantifolia* (Christm.) Swingle	Lime	• Fruit hydroalcoholic extract (containing 4%–6% of synephrine) reduced food consumption and body weight gain in rats [101]
			• Peel extract reduced body weight gain, insulin, and leptin and upregulated lipid-metabolizing enzymes in rats fed an HFD [102]
			• Lime essential oil led to decreased body weight and food intake in ketotifen-induced obesity in mice [103]

23	*Citrus limon* (L.) Burm. f.	Lemon	• Leaf extracts inhibited pancreatic lipase in vitro [104]
			• Lemon essential oil increased lipolysis and suppressed body weight gain in rats [105]

24	*Citrus paradise* Macfad.	Grapefruit	• Aqueous extracts suppressed hepatic tissue fat accumulation and lipogenesis and increased β-oxidation in obese rats [106]
			• Fruit produced weight reduction in obese rats [107]
			• Fruit resulted in weight loss in patients [108]

25	*Cochlospermum tinctorium* Perrier ex A.Rich.		None reported

26	*Cocos nucifera* L.	Coconut	• Coconut oil promoted a reduction in abdominal obesity in women [109]; reduced fat accumulation in adipocytes during its differentiation [110]
			• Coconut husks inhibited pancreatic lipase in vitro [111]
			• Coconut water prevented obesity in Wistar rats fed an HFD [112]
			• Coconut water vinegar reduced the body weight, fat mass, expression of SREBP-1, RBP-4, and resistin and elevated expression of adiponectin in mice fed HFD [113]

27	*Commiphora africana* (A.Rich.) Endl.	African silk tree	None reported

28	*Cucumis sativus* L.	Cucumber	• Methanol extract of mesocarp contained saponins that are active pancreatic lipase inhibitors [114]
			• Extracts of fruits were shown to have antiadipogenic activity in rats [115]

29	*Cucurbita ficifolia* Bouché	Pumpkin	• Chloroform extract attenuated adipogenesis in human mesenchymal stem cells [116]
			• Aqueous fruit extract modulated systemic chronic inflammation in MSG-induced obesity in mice [117]
			• Ethanol fruit extract reduced body weight in obese rats fed an HFD [118]

30	*Cucurbita pepo styriaca* L.	Pumpkin	• Methanol leaf extracts decreased food intake and BMI in progesterone-obese mice [119]

31	*Curcuma longa* L.	Turmeric	• Curcumin prevented adipogenesis in 3T3-L1 adipocytes, obesity, and angiogenesis in C57/BL mice [120]
			• Rhizomes reduced body weight and white adipose tissue, suppressed adipocyte differentiation and lipogenesis, increased lipolysis and β-oxidation in HFD fed obese rats [121], and inhibited pancreatic lipase in vitro [104]
			• Fermented 50% ethanol extract suppressed body weight, white adipose tissue weight, adipocyte differentiation, and lipogenesis in HFD-induced obese rats [121]
			• Ethanol extract prevented fat buildup and insulin resistance in diabetic obese db/db mice [122]
			• Fermented ethanol extract suppressed body weight and fat mass and increased lipolysis in the C57BL/6J ob/ob mouse model [123]
			• Ethyl acetate fraction inhibited lipogenesis and enhanced lipolysis in differentiated 3T3-L1 cells [124]
			• Ethanol extract of turmeric rhizomes showed antiadipogenesis potential in HepG2 cells [125]
			• Methanol and aqueous extracts prevented lipid accumulation in 3T3-L1 fat cells [47]

32	*Cymbopogon citratus* (DC.) Stapf	Lemon grass	• Citral, a major component in lemongrass oil, increased energy dissipation and reduced lipid accumulation in rats [126]

33	*Cyperus esculentus* L.	Tiger nut, yellow nutsedge	• The defatted extract reduced weight gain and adipose tissue weight in obese mice as well as adipocyte size [127]

34	*Detarium senegalensis* J.F.Gmel.	Sweet datar, wild cassia	None reported

35	*Fragaria vesca* L.	Strawberry	None reported

36	*Garcinia kola* Heckel.	Bitter kola	None reported

37	*Glycine max* (L.) Merr.	Soy beans	• Genistein inhibited adipocyte differentiation in ovariectomized mice [128]
			• Anthocyanins in black soybean seed coats decreased body weight, fat mass, and lipid profile in HFD-fed rats [129]; decreased fat accumulation and lipogenesis in DIO mice [130]
			• Ethanol extract from seeds inhibited adipogenesis in 3T3-L1 cells [131]
			• 7,3′,4′-Trihydroxyisoflavone reduced lipid content and adipocyte differentiation in 3T3-L1 fat cells and reduced body weight in C57BL/6 mice fed an HFD [132]
			• Soybean protein isolate reduced weight gain and adipose tissue mass, attenuated hepatic steatosis, and increased GLP-1 secretion in mice fed HFD [133]
			• Black soybean seed coat extract suppressed fat accumulation, enhanced energy expenditure, and suppressed inflammation in mice fed an HFD [134]
			• Unhulled soybean extract exhibited a weight loss effect in rats placed on high-fat high-sucrose diet [135]
			• Unripe soybean leaves reduced the accumulation of adipose tissue and increased lipid metabolism in HFD fed mice [136]

38	*Gnetum africanum* Welw.	Okazi, Afang	None reported

39	*Gongronema latifolium* Benth.	Bush buck, Utazi	None reported

40	*Guiera senegalensis* J.F.Gmel.	Moshi medicine	None reported

41	*Heinsia crinita* (Afzel.) G.Taylor	Bush apple	None reported

42	*Hibiscus sabdariffa* L.	Roselle	• Aqueous extract reduced fat accumulation in the livers of hamsters fed an HFD [137]
			• Aqueous calyx extract reduced body weight in rats [138]; reduced body weight gain in MSG-obese mice [139]; reduced body weight and abdominal fat in HFD obese rats [140]; reduced body weight and leptin in obese rats with myocardial infarction [141]; decreased body weight gain, hepatic steatosis, and adipocyte hyperplasia in high-fructose, high-fat diet-fed rats [142]; decreased body weight; and attenuated hepatic steatosis via decreased expression of SREBP-1c and PPAR-γ in DIO in mice [143]
			• Methanol and aqueous flower extract displayed pancreatic lipase and α-amylase inhibitory activity in vitro [42]
			• Aqueous flower extract reduced obesity and abdominal fat, while attenuating liver steatosis in humans with BMI ≥ 27 [144]; inhibited lipid deposition and adipogenic transcription factors expression of 3T3-L1 fat cells [145]; inhibited adipocyte differentiation in 3T3-L1 cells via the PI3-K and MAPK pathway [146]
			• Ethanol calyx extract prevented body weight increase in rats [147]
			• Sorrel methanol extracts inhibited the activities of pancreatic lipase, α-glucosidase, and α-amylase and enhanced reduction of lipid deposition and elevated lipolysis of 3T3-L1 cells [148]
			• Habisic acid and 6-methyl ester from 50% methanol and acetone *H. sabdariffa* extract inhibited α-amylase activity [149]
			• Polyphenolic extract suppressed adipogenesis in 3T3-L1adipocytes and inhibited hepatic lipogenesis in obese hamsters [150]
			• Dried ground calyces reduced body weight, adiposity, and proinflammatory cytokines in WAT and increased plasma GLP1 and IL-6 levels in brown adipose tissue in mice fed an HFD [151]
			• A combination extract of *H. sabdariffa* and *Lippia citrio* polyphenols decreased obesity-related symptoms in overweight/obese subjects [152]

43	*Ipomoea batatas* (L.) Poir.	Sweet potato	• Aqueous tuber extract decreased food consumption and body weight gain in normal rats [153]
			• Sweet purple potato anthocyanin-rich extract had antilipogenic and lipolytic effects in 3T3-L1 adipocytes [154]; reduced body weight gain and fat weight and preserved the leptin signaling capability in rats fed HFD [136]

44	*Irvingia gabonensis* (Aubry-Lecomte ex O'Rorke) Baill.	Wild mango	• Extracts reduced body weight in overweight participants [155]
			• A combination of *Cissus quadrangularis* and *Irvingia gabonensis* reduced body weight, body fat, and waist size of obese or overweight participants [156]
			• Seed extract repressed adipogenesis in adipocytes [157]; reduced body weight and body fats of obese subjects [158, 159]
			• Stem bark aqueous extracts reduced pancreatic lipase activities in normal rats [160]

45	*Khaya senegalensis* (Desv.) A.Juss.	African mahogany	• Aqueous bark infusion inhibited porcine pancreatic lipase activity in vitro [161] and decreased body weight and abdominal lipid content of rats fed HFD [162]

46	*Luffa aegyptiaca* Mill.	Luffa	None reported

47	*Lycopersicon esculentum* Mill.	Tomato	• Tomato vinegar beverage reduced body weight, visceral fat, and insulin resistance via AMPK/PPARα in DIO in mice [163]
			• The ingestion of raw tomato before a meal declined body weight and fat mass in female subjects [164]

48	*Mentha pulegium* L.	Mint leaf	None reported

49	*Momordica balsamina* L.	Balsam apple, African cucumber	None reported

50	*Momordica charantia* L.	Bitter melon	• Methanol seed extracts were shown to have pancreatic lipase activity [100]
			• Freeze-dried bitter melon juice reduced body weight, visceral fat mass, insulin, and leptin in rats fed an HFD [165, 166]; reduced insulin resistance as well as obesity-associated macrophage and mast cell infiltration in fat tissues in obese mice [167]; displayed reduced adiposity and enhanced lipid oxidative enzyme activities and UCP-1 in rats fed an HFD [168]
			• The fruit n-butanol fraction inhibited lipase in vitro [169]; decreased the weight of epididymal WAT and visceral fat, and decreased the mRNA levels of adipose leptin and resistin in mice on HFD [170]
			• Aqueous extract of the whole fruit suppressed body weight and insulin levels in obese mice [171]
			• Ethanol fruit extract inhibited lipase in vitro [172]; inhibited lipoprotein lipase and proliferation of 3T3-L1 preadipocytes [173]
			• Aqueous and ethanol de-seeded fruit extract decreased body weight, visceral tissue weight, and insulin levels in mice fed HFD [174]

51	*Moringa oleifera* Lam.	Moringa, drumstick tree, miracle tree	• Leaf extract induced an antiobesity effect in rats fed an HFD [175]; reduced inflammation and lipid accumulation and induced thermogenesis in human adipose-derived mesenchymal stem cells [176]
			• Ethanol leaf extract reduced body weight and downregulated leptin and resistin mRNA expression, but adiponectin gene expression was upregulated in obese rats [177]; prevented nonalcoholic fatty liver disease in obese mice [178]
			• Leaf powder decreased food intake and body weight in HFD obese rats [179]
			• Methanol leaf extract decreased body weight in rats fed an HFD [175]
			• Leaves reduced body weight, BMI, and insulin in T2D subjects [180]
			• Ethanolic seed extract reduced lipid parameters, body weight, and adipose tissue mass [86]

52	*Mucuna flagellipes* Hook.f.	Ukpo	• Ethanol leaf extract and n-hexane fraction inhibited pancreatic lipase [181]

53	*Murraya koenigii* (L.) Spreng.	Curry leaf	• Dichloromethane, ethyl acetate, and methanol leaf extracts exhibited lipase inhibitory activity in vitro [182]
			• Dichloromethane and ethyl acetate leaf extract as well as an isolated compound, mahanimbine, reduced body weight in rats fed an HFD [183]
			• Ethanol leaf extract reduced body weight in obese rats [184]

54	*Myristica fragrans* Houtt.	Nutmeg	• Ethanol mace extract indicated pancreatic lipase inhibitory activity [185] and reduced food consumption and body weight in obese rats [186]
			• Ethanol extract of kernel stimulated AMPK in differentiated C2C12 cells and diminished food intake, body weight gain, and epididymis fat in mice fed an HFD [187]

55	*Ocimum basilicum* L.	Basil	• Methanol leaf extract inhibited pancreatic lipase activity in vitro [188] and leaf extracts inhibited adipogenesis in 3T3-L1 adipocytes [63]

56	*Ocimum gratissimum* L.	Scent leaf, fever plant, fever bush	• Aqueous leaf extract reduced body weight and adipocytes in ovariectomized rats [189]
			• Methanol leaf extract inhibited pancreatic lipase activity in vitro [188]

57	*Persea americana* Mill.	Avocado pear	• Methanol extracts from fruits inhibited acetyl-CoA carboxylase activity in vitro [190]
			• Hydroalcohol fruit extract decreased FAS, HMG-CoA reductase, and accumulation of fatty droplets in the liver, increased lipoprotein lipase activity in rats fed an HFD [191], and reduced BMI and leptin in rats fed an HFD; furthermore, the mRNA expression of FAS, lipoprotein lipase, and leptin in subcutaneous and visceral adipose tissue were reduced [192]; regulation of obesity-related genes in rats fed an HFD [193]
			• Aqueous and methanol leaf extracts decreased the body weight gain of rats fed a hyperlipidemic diet [194]

58	*Piper guineense* Schumach. & Thonn.	Benin pepper, Uziza seeds, Ashanti pepper	None reported

59	*Piper nigrum* L.	Black pepper	• Piperine diminished fat accumulation and body weight of obese rats [195]
			• Hexane, ethylacetate, ethanol, and aqueous seed extracts as well as piperine suppressed body weight and fat mass, modulated lipid metabolic enzymes, and improved insulin and leptin sensitivity in DIO in rats [196, 197]
			• Methanol seed extract and piperine inhibited adipogenesis by decreasing PPARγ activity in 3T3-L1 cells [198]
			• Piperonal decreased body weight, fat percentage, adipocyte size, and expression levels of adipogenic genes in obese rats [199]

60	*Prosopis africana* (Guill. & Perr.) Taub.	False locust	None reported

61	*Psidium guajava* L.	Guava leaves	• Leaf extract inhibited pancreatic lipase [64, 104]
			• Ethanol leaf extract inhibited differentiation of 3T3-L1 preadipocytes [200]
			• Phenolic compounds from leaves improved insulin resistance in mice fed an HFD [201]
			• Leaf powder supplementation of the HFD feed resulted in decreased fat deposition in obese rats [202]
			• Aqueous leaf extract enhanced insulin sensitivity and increased serum adiponectin content, AMPK, and PPARs in both liver and skeletal muscle tissues in mice fed a high-fructose-high-fat diet [203]
			• Pink guava puree reduced body weight in rats fed an HFD [204]

62	*Pterocarpus mildbraedii* Harms	Ora, Oha	• Ethanol leaf extract and n-hexane fraction inhibited pancreatic lipase [181]

63	*Senna occidentalis* (L.) Link	Coffee senna	None reported

64	*Spinacia oleracea* L.	Spinach	• Flavonoid-rich extracts possessed appetite-suppressing effects by inducing a quicker than normal release of cholecystokinin in rats [205, 206]
			• Antioxidant-rich extract of leaf decreased insulin levels and insulin resistance in rats with metabolic syndrome [207] and decreased food intake, weight gain, and pancreatic lipase activity in rats fed an HFD [208]
			• Chlorophyll-rich extract declined body weight and fat mass gain in mice fed an HFD [209]

65	*Tamarindus indica* L.	Tamarind	• Pulp aqueous extract had antiobesity effects in HFD-induced obesity rats [210]
			• Methanol and aqueous husk extract displayed pancreatic lipase and α-amylase inhibitory activity in vitro [42]
			• Aqueous pulp extract decreased body weight of rats [211]
			• The protein fraction of seeds decreased food intake and plasma leptin in obese Wistar rats [212]
			• Methanol extract of seed coat reduced body weight and adiposity, improved insulin resistance index, and reversed fatty liver in rats fed an HFD [213]
			• Ethanol extract decreased body weight in cafeteria diet and sulpiride-induced obese rats [214]
			• Trypsin inhibitor isolated from tamarind seeds decreased leptin in obese Wistar rats [215] and reduced food ingestion and body weight in rats placed on standard protein diet [216]
			• Aqueous extract decreased plasma leptin, FAS activity, and hepatic steatosis in obesity-induced rats [210]

66	*Tetrapleura tetraptera* (Schumach. & Thonn.) Taub.	Aiden pod	• Aqueous stem bark extract decreased body weight, fat weight, and food ingestion in DIO obesity in rats [217]
			• Hydroethanolic extract reduced weight gain, insulin levels, and insulin resistance and increased adiponectin in high-carbohydrate, HFD obese and T2D rats [218]

67	*Vernonia amygdalina* Delile	Bitter leaf	• Leaf supplementation decreased body weight and WAT in HFD-induced obesity Wistar rats [219]
			• Aqueous and methanol leaf extracts induced weight loss in rats fed an HFD [220] and reduced appetite via modulation of appetite regulatory hormones [221]
			• Bitter leaf decreased body weight, adipose tissue weights, and fat deposits in the liver of obese rats fed an HFD supplemented with bitter leaf [222]

68	*Vigna subterranea* (L.) Verdc.	Bambara groundnut	None reported

69	*Zingiber officinale* Roscoe	Ginger	• Aqueous rhizome extracts declined body weight, fat mass, and leptin in obesity diabetic rats [88]
			• Methanol and aqueous rhizome extract displayed pancreatic lipase and α-amylase inhibitory activity in vitro [42]
			• Aqueous rhizome extract reduced body weight gain, insulin, and leptin in obese rats [223]; decreased body weight, body fats, and leptin in obese diabetic rats [224]; inhibited in vitro pancreatic lipase activity and reduced body weight and fat weights in HFD fed mice [225]; reduced body weight, fat weight, and number and size of fat vacuoles in HFD obese rats [226]; decreased body weight gain, Lee's index, and adipose tissue weight; and enhanced thermogenesis and lipid homeostasis in both preventive and ameliorative studies using HFD-induced obese rats [227]
			• Rhizome powder included in the diet led to a reduction in body weight in albino rats fed an HFD [228]; decreased body weight, BMI, waist and hip circumferences, body composition, and total appetite score and increased thermogenesis in obese women [229]; decreased hunger and food intake and increased fullness in men who were overweight [230]; decreased weight gain in high-fat high-carbohydrate diet-fed rats [231]
			• Ethanol extract of rhizomes reduced body weight in rats fed an HFD [232]; reduced body weight, insulin, free fatty acids, and phospholipids in rats fed an HFD [233]; reduced body weight and inhibited liver steatosis by regulating the expressions of hepatic genes associated with lipogenesis and lipolysis in mice fed an HFD [234]; reduced weight gain and fat accumulation and increased energy expenditure [235]
			• Methanol and ethyl acetate rhizome extracts reduced body weight and insulin in gold thioglucose-induced obesity in mice [236]
			• High-hydrostatic pressure extract of ginger decreased body weight and white adipose tissue mass with an increase in fecal lipid excretion via regulation of microRNA-21/132 expression and AMPK activation in white adipose tissue of rats fed a HFD [237]
			• Ethyl acetate soluble portion of rhizome inhibited pancreatic lipase activity [238]
			• Gingerol decreased bodyweight, leptin, insulin, amylase, and lipase in HFD obese rats [239]; decreased weight gain, fat accumulation, insulin, and leptin in HFD fed rats [240]; decreased weight gain and adipose tissue and increased expression of fatty acids' beta-oxidation enzymes in mice fed a HFD [241]; decreased liver weight and insulin levels in mice fed a HFD [242]; inhibited adipogenesis and decreased expression of various adipogenic/lipogenic marker proteins, thereby enhancing lipolysis in 3T3-L1 preadipocytes [243–245]
			• Galanolactone isolated from ginger suppressed adipocyte differentiation and lipid droplet accumulation and decreased expression of adipogenic transcription factors and adipogenic marker genes in 3T3-L1 preadipocytes [246]
			• Zingerone increased isoprenaline-induced lipolysis in adipocytes of rats fed a NPD and HFD [247]
			• Ginger essential oil decreased weight gain, serum free fatty acid, and hepatic lipid accumulation in mice fed a HFD [248]
			• Gingerenone A decreased weight gain and adipocyte size and free fatty acid levels and regulated fatty acid metabolism and mitochondrial biogenesis via activation of AMPK in adipose tissue in mice fed a HFD [249]

70	*Ziziphus jujube* Mill.	Jujube fruit	• Ethanol fruit extract inhibited porcine pancreatic lipase activity in vitro [250]
			• Powder decreased BMI, fat percentage, and body weight in healthy men and women [251]
			• Methanol leaf extract reduced body weight and increased body temperature in rats fed a HFD [252]
			• Fruit extract and chloroform fraction inhibited adipogenesis by decreasing the expression of PPARγ, C/EBPα, and β in 3T3-L1 preadipocytes [253]

*Note:* CB1, cannabinoid receptor type 1; ED_50_, median effective dose; HepG2, human hepatoma; HMG-CoA, β-hydroxy β-methylglutaryl-CoA; IC_50_, half maximal inhibitory concentration; LC_50_, median lethal concentration; LD_50_, median lethal dose; LLC-MK2, rhesus monkey kidney epithelial cells; SREBP-1:sterol regulatory element–binding transcription factor 1; TRPV-1, transient receptor potential cation channel subfamily V member 1.

Abbreviations: AMP, adenosine monophosphate; AMPK, adenosine monophosphate–activated protein kinase; BMI, body mass index; C/EBP, CCAAT/enhancer binding proteins; CoA, coenzyme A; DIO, diet-induced obesity; FAS, fatty acid synthase; GLP-1, glucagon-like peptide-1; HCD, high-carbohydrate diet; HFD, high-fat diet; HSL, hormone-sensitive lipase; IL-6, interleukin 6; LDH, lactate dehydrogenase; LPL, lipoprotein lipase; MAPK, mitogen-activated protein kinase; mRNA, messenger RNA; MSG, monosodium glutamate; NAFLD, nonalcoholic fatty liver disease; NOAEL, no observed adverse effect level; NPD, normal pellet diet; PI3-K, phosphoinositide 3-kinases; PPAR, peroxisome proliferator–activated receptor-γ; RBP-4, retinol binding protein 4; T2D, type 2 diabetes; TNF, tumor necrosis factor; UCP2, uncoupling protein 2; WAT, white adipose tissue.

**Table 4:** Reports of toxicity evaluation of plants used to treat obesity.

S/N	Botanical name	Common name	Plant toxicity (dose, organs, plant part, and animal/in vitro studies
1	*Acacia nilotica* (L.) Delile	Gum arabic tree; Scorpion mimosa	• Pods showed low toxicity potential by decreasing body weight and hemoglobin in rats fed a 2% and 8% *Acacia* content diet for 2 and 4 weeks [254]
			• Aqueous root extract induced hepatotoxicity at doses > 250 mg/kg bw when fed to rats for 4 weeks [255]
			• Aqueous stem bark extract resulted in a slight decrease in body and organ weight and impaired biochemical parameters when fed to mice for 28 days at 1 g/kg bw [256]

2	*Adansonia digitata* L.	Baobab	• Methanol leaf extracts had an LD_50_ > 2000 mg/kg in rats [257]
			• Petroleum ether, ethanol, and aqueous stem bark extracts indicated no toxicity at 5000 mg/kg in rats [258]
			• LD_50_ was > 2900 mg/kg for the crude seed extract [259]
			• Fruit extract was shown to have LD_50_ > 5000 mg/kg in rodents [260]

3	*Allium cepa* L.	Onions	• Methanol extract of scales and flesh administered to mice had LD_50_ > 200 mg/kg p.o. [261]
			• Leaf extracts indicated LD_50_ > 2000 mg/kg p.o. in mice [262]
			• The single oral dose of aqueous extracts in rats indicated LD_50_ > 5000 mg/kg [263]

4	*Allium sativum* L.	Garlic	• Methanol extract of scales and flesh had LD_50_ > 200 mg/kg in mice [261]
			• Subacute toxicity studies of aqueous bulb extract fed to rats for 4 weeks p.o. indicated safety at 1200 mg/kg [264, 265], LD_50_ was > 5000 mg/kg bw p.o in rats [265], and LC_50_ of the aqueous extract in juvenile *C. carpio* (fish) was ∼ 253.19 mg/L [266]

5	*Aloe barbadensis* Mill; *Aloe vera* (L.) Burm.f.	Aloe vera	• Gel and the low molecular weight fraction of leaves promoted cellular damage in vitro [267]
			• Methanol extract of the gel had LD_50_ ≥ 16 g/kg bw in rats with no signs of toxicity being noted in subacute studies for 6 weeks [268]
			• Hydroalcoholic extracts of leaves indicate oral acute LD_50_ ≥ 5120 mg/kg and subchronic LD_50_ > 640 mg/kg in chicks [269]
			• Dried leaf extract had LC_50_ of 3.59 μg/mL and 120.65 mg/kg p.o in brine shrimp and mice respectively [270]
			• Leaf powder (2, 4, and 8 g/kg bw) fed to rats for 90 days (subchronic toxicity) indicated low toxicity in rats fed 8 g/kg [271]
			• Gel extract administered orally at 150 mg/kg daily for 8 weeks indicated slight toxicity in the reproductive parameters of rats [272]
			• A single case of toxicity in humans has been reported [273]

6	*Alstonia boonei* De Wild.	Cheese wood	• Ethanol extract of stem bark and leaves (acute toxicity study) had LD_50_ of 5000 mg/kg oral single dose in rats [56, 274, 275] and mice [276]
			• Aqueous extract of stem bark indicated possible toxicity in doses > 500 mg/kg orally in rats after 4 weeks [277], but using the same concentration and route no toxicity was found in mice [276]
			• Extract and fractions (n-hexane, chloroform, ethylacetate and aqueous) induced hepatotoxicity and nephrotoxicity at 400 mg/kg bw when fed to rats for 21 days [278]

7	*Ananas comosus* (L.) Merr.	Pineapple	• Hydroalcoholic leaf extract had an LD_50_ > 3000 mg/kg p.o. in rats [279], and a similar finding was found in mice administered methanol leaf extract [280]
			• Leaf extract showed no toxicity in rats fed 5000 mg/kg nor 1000 mg/kg in acute and subacute studies, respectively [281]
			• Fruit aqueous of fruits had an LD_50_ > 2000 mg/kg p.o. in mice [282]

8	*Anthocleista vogelii* Planch.	Cabbage tree	• Aqueous extract stem bark revealed no toxicity at 16 g/kg single dose and 1000 mg/kg after being fed to rats for 4 weeks [283]
			• Acute toxicity studies in rodents indicated LD_50_ > 3200 mg/kg p.o. for methanol leaf extract [284, 285], 5000 mg/kg p.o. for ethanol root extract [286], and 6400 mg/kg p.o. for ethanol root bark extract [61]
			• Petroleum ether leaf extract had an LD_50_ of 2000 mg/kg i.p. in mice [287]
			• Subacute toxicity studies of methanol leaf extract indicated no toxicity up to 800 mg/kg in rats [284]

9	*Azadirachta indica* A. Juss.	Neem tree	• Ethanol stem bark extract indicated low toxicity in rats after 21 days of oral administration (300 mg/kg) [288]
			• Oil had an LD_50_ of 31.95 g/kg and no significant toxicity was observed at doses < of 1600 mg/kg/day in subacute studies (28 days) [289]; liver damage was noted in rats fed 1600 mg/kg/day oil after 90 days [290]
			• Methanol flower extract had LD_50_ > 12 g/kg bw in rats, whereas subacute toxicity test at 750 mg/kg bw and above revealed low toxicity after 90 days [291]
			• LC_50_ relative toxicity factor of leaf methanol extract was 431.931 mg/L after 96 h exposure in fish [292]

10	*Beta vulgaris* L.	Beetroot	• Aqueous and methanol extracts showed no toxicity at 2000 mg/kg oral dose in mice [293, 294]
			• LD_50_ > 2000 mg/kg oral dose in rats for ethanol, methanol, and chloroform extracts [295]
			• LD_50_ of aqueous extracts was > 5000 mg/kg oral dose in rats, whereas subacute studies revealed possible toxicity at doses > 3000 mg/kg p.o. after 28 days [296]
			• No evidence of genotoxicity was induced by extracts or juice in human C3A liver cells [297]
			• Cytotoxicity of red beetroot extract was noted in cancer cells [298]

11	*Boscia senegalensis* Lam.	Arabic	• Leaves and fruits are not toxic to insects [299–301]

12	*Brassica oleracea* L.	Cabbage	• Ethanol leaf extracts were not toxic at 5000 mg/kg (single dose) and indicated hepatoprotective activity at 300 and 500 mg/kg when fed to rats [302]
			• LD_50_ of ethanol whole plant extracts was > 4000 mg/kg p.o. in rats and no toxicity was observed at 800 mg/kg after 28 days in subacute studies [303]
			• In mice, ethanol leaf extract was found to be safe at 2000 mg/kg oral dose after being fed to mice for 4 weeks [304]

13	*Brassica nigra* (L.) K.Koch	Black mustard seed	• Aqueous seed extract had an LD_50_ > 4000 mg/kg oral dose in rats [305]
			• Methanol leaf extract was nontoxic at ≤ 400 mg/kg in rats fed the extract for 21 days [306]

14	*Capsicum annuum* L.	Chili pepper, cayenne pepper	• LD_50_ for the aqueous and ethanol extract was 12,043 mg/kg and 5492 mg/kg, respectively in mice [307]

15	*Capsicum chinense* Jacq.	Adjuma, Ají Dulce	• Ethanol fruit extracts had an LC_50_ of 39.7 ± 2.1 μg/mL in zebrafish embryos [308]
			• Methanol fruit extract (200 mg/kg bw) induced genotoxicity in bone marrow cells [309]

16	*Carica papaya* L.	Pawpaw	• Methanol leaf extracts indicated cytotoxic activity at LC_50_ of 118.73 μg/mL in brine shrimp [310]; LD_50_ > 3200 mg/kg p.o. in mice, and in the subacute studies, no signs of toxicity were noted up to 3200 mg/kg/day after 60 days [311]
			• Aqueous extract of the unripe fruit had an LD_50_ of 2520 mg/kg (acute oral toxicity study) in rats; chronic toxicity studies showed no toxic effects after daily treatment with doses up to 250 mg/kg oral dose [312]
			• Aqueous leaf extract had LD_50_ > 5000 mg/kg, and in subacute studies, no toxicity was noted up to 500 mg/kg p.o. in rats [313]
			• Leaf extract had an LD_50_ > 2 g/kg [314, 315], leaf extract had LD_50_ > 5000 mg/kg p.o. in mice [316]
			• Methanol extract of seeds showed signs of hepatotoxicity at 200 mg/kg p.o. in rats [317]
			• Oral intake of leaf extract by rats at 0.01–2 g/kg/day for 28 days showed no toxicity [318]

17	*Ceratotheca sesamoides* Endl.	False sesame	• Methanol leaf extract had an LD_50_ > 3000 mg/kg oral dose in mice [319]

18	*Chrozophora senegalensis* (Lam.) A.Juss. ex Spreng.		• Aqueous leaf extract had an LD_50_ > 5000 mg/kg oral dose in rats [320]
			• Methanol whole extract had an LD_50_ of 175 mg/kg i.p. in mice [321]
			• Acetone stem extract and petroleum ether stem and leaf extract showed negligible toxicity at 100 μg/mL in Vero cells; no toxicity was noted after 4 consecutive days treatment with 500 mg/kg oral dose of the aqueous leaf extract in mice; toxicity was noted when administered at 100 mg/kg i.p. [322, 323]

19	*Cinnamomum verum* J.Presl	Cinnamon	• Aqueous extract had an LD_50_ > 2.0 g/kg oral dose in rats [324]
			• Aqueous bark extract had an LD_50_ > 5000 mg/kg p.o. and extract was safe at doses of 200 mg/kg administered p.o. after 4 weeks in rats [325]

20	*Citrullus colocynthis* (L.) Schrad	Bitter lemon, bitter apple, egusi melon, wild gourd	• LD_50_ of the fruit extract was 162.4 mg/kg p.o. in rats; mild necrotic reaction was noted at doses of 200 and 400 mg/kg after administration for 10 weeks [326]
			• Both pulp and seed extracts were toxic at 200 mg/kg p.o. to rabbits after 4 weeks [327]
			• Ethanol extract of fruits had LD_50_ of 100 mg/kg p.o. in rats; subacute studies revealed adverse effects on liver and kidney at 12.5 and 25 mg/kg when administered twice per week for 8 weeks [328]
			• LD_50_ of the methanol leaf extract was 1311.45 mg/kg p.o. in rats; hepatorenal toxicity was revealed at 265 and 440 mg/kg after 6 weeks [329]
			• Alcoholic extract of seeds was toxic to the liver at concentrations ≥ 200 mg/kg when administered i.p. after 14 days to rats [330]
			• Aqueous extract given orally to rats at 50 and 100 mg/kg was not found to be toxic after 28 days of treatment [331]

21	*Citrullus lanatus* (Thunb.) Matsum. & Nakai	Watermelon	• Aqueous fruit peel extract had LD_50_ > 3000 mg/kg oral dose in mice [332, 333]
			• Seed oil was safe at a single oral dose of 2000 mg/kg in rats [334, 335]
			• Chloroform fruit extract had an ED_50_ 241.29 μg/mL in brine shrimp, whereas the butanol and ethyl acetate extracts were not toxic [336]
			• Aqueous seed extract had an LD_50_ > 2000 mg/kg p.o. in rats [337]
			• No mortality was observed in rats given seed extract orally up to 5 g/kg [338]

22	*Citrus aurantifolia* (Christm.) Swingle	Lime	• LC_50_ values of methanol, ethyl acetate, and aqueous leaf extracts in brine shrimps were ∼150, 377, and 587 μg/mL, respectively [339]
			• LD_50_ of stem bark methanol extract was 5000 mg/kg oral dose in rats [340]
			• Aqueous root extract had an LD_50_ ≥ 5000 mg/kg p.o. (single dose) in rats with signs of toxicity noted at 1200 mg/kg after 90 days of oral administration [341]
			• Leaf methanol extract had LD_50_ = 3280 mg/kg i.p. in mice [342]
			• Essential oil exhibited no toxicity in acute toxicity tests; however, in subchronic studies, mild toxicity was observed at 100 and 500 mg/kg/day in rats after 60 days [343]

23	*Citrus limon* (L.) Burm. f.	Lemon	• Hydroalcoholic extract of peel did not show signs of toxicity at 2000 mg/kg single oral dose in rats [344]
			• LD_50_ of aqueous methanol peel extract was > 5000 mg/kg in rats via oral administration [345]
			• In a subacute toxicity study, fruit juice was considered nontoxic to rats [346]
			• No toxicity was noted after treatment with 500 mg/kg of essential oils in mice, but possible cytotoxicity was noted in other cells [347]
			• Ethyl ether extract of peel showed toxicity in two fruit fly species [348]

24	*Citrus paradise* Macfad.	Grapefruit	• Fruit juice resulted in hepatotoxicity at 400 mg/kg p.o and above in rats after 60 days; no toxicity was noted at ≥ 3000 mg/kg p.o. after single dose [349]
			• Ethyl ether extract of peel showed toxicity in two fruit fly species [348]
			• Grapefruit peel oil indicated toxicity and larvicidal activity against *Aedes aegypti* [350]
			• Grapefruit seed extract exhibited toxicity in NIH-3T3 cells and connective tissue in rats [351]
			• LD_50_ of aqueous fruit pulp extract was > 5000 mg/kg p.o. in rats [352]
			• In acute toxicity studies, no mortality was observed up to 2000 mg/kg p.o. single dose of methanol leaf extract in mice [353]

25	*Cochlospermum tinctorium* Perrier ex A. Rich.		• Tubercle essential oil extracts were toxic in leukemia K562 cells with an IC_50_ values of 80 pg/mL; the essential oil extract from the central cylinder was less toxic with IC_50_ of 1600 pg/mL [354]
			• LD_50_ values of the aqueous methanol extracts of leaf, root, and root bark were 118.32, 288.53, and 288.53 mg/kg, respectively, when administered to mice i.p. [355]

26	*Cocos nucifera* L.	Coconut	• In acute toxicity study of aqueous green coconut husk fiber and butanol extracts, no toxicity was noted at 3000 mg/kg p.o. in mice, but lethality emerged at 500 and 700 mg/kg i.p, and subacute studies revealed low toxicity [356]
			• Ethyl acetate fraction of the root water extract indicated no toxicity at 2000 mg/kg oral single dose or subchronic oral toxicity at daily doses of 200 mg/kg bw for 28 days [357]
			• LD_50_ of ethyl acetate soluble proanthocyanidins was > 2000 mg/kg p.o. and the oral daily administration for 28 days was safe up to 14 mg/kg in rats [358]
			• LD_50_ of chloroform, petroleum ether, and methanol leaf extracts was ≥ 2000 mg/kg p.o. in mice, and no signs of toxicity were observed after oral daily administration of these extracts at 200 mg/kg for 4 weeks [359]
			• Rats treated with fermented virgin coconut oil displayed no toxicity signs at 5000 mg/kg in acute and < 2000 mg/kg p.o. in subchronic and chronic toxicity studies of 28 and 90 days, respectively, in rats [360]
			• Aqueous leaf extract showed organ toxicity at 2000 mg/kg/day i.m. in mice after 4 days [361]
			• Methanol endocarp extract showed no signs of toxicity and did not result in mortality in mice at 5000 mg/kg p.o. single dose [362]

27	*Commiphora africana* (A. Rich.) Endl.	African silk tree	• LD_50_ of ethanol extract root was ≥ 5000 mg/kg oral dose in mice [363]
			• Ethanol leaf extract was safe up to 150 mg/kg when given as an oral daily dose to mice for 10 days [364]
			• The stem bark and whole stem extracts were nontoxic to LLC-MK2 monkey kidney epithelial cells with CC_50_ > 30 μg/mL [365]
			• LD_50_ of hydroethanolic stem bark extract was 3708.7 mg/kg i.p and 471.2 mg/kg i.p. in rats and mice, respectively [366]

28	*Cucumis sativus* L.	Cucumber	• Fruit homogenate administered to mice up to 5 mL/kg p.o. was safe [367]
			• Ethanol fruit extract had an LD_50_ ≥ 2000 mg/kg oral dose in rats [368]
			• Single dose of aqueous fruit extract was well tolerated without mortality at 2000 mg/kg; also, repeated doses of 1000 mg/kg within 90 days in rats were not toxic [369]
			• Water/ethanol fruit extract indicated no toxicity < 2 mg/mL in porcine aortic endothelial cells [370]

29	*Cucurbita ficifolia* Bouché	Pumpkin	• LD_50_ of freeze-dried juice was 625 mg/kg intraperitoneally and 3689 mg/kg orally in mice [371, 372]

30	*Cucurbita pepo styriaca* L.	Pumpkin	• No toxicity signs were noted in rats orally administered seed extract of 2000 mg/kg [373]
			• Seed extract LD_50_ ≥ 5000 mg/kg oral dose in rats and was found safe < 1000 mg/kg/day after 8 days of administration [374]
			• Seed oil was safe at 2000 mg/kg p.o. single dose and at 1000 mg/kg repeated dose in subacute and subchronic studies for 28 and 90 days, respectively, in rats [375]; indicated cytotoxicity against breast carcinoma cells (MCF7) with IC_50_ of 0.40–1.01 mg/mL [376]
			• Hydroalcoholic leaf extract showed low cytotoxicity (IC_50_ ≥ 132 μg/mL) in CHO, fibroblast, CT26, and HepG2 cells [377]

31	*Curcuma longa* L.	Turmeric	• NOAEL was 1000 mg/kg bw/day after 90-day administration to rats [378]
			• Ethanol rhizome extract had LD_50_ > 5000 mg/kg oral dose in rats; subchronic oral administration of the extract did not induce toxicity to the animals [379]
			• Essential oils were nontoxic at 560 mg/kg/d after 28-day administration to rats [380]
			• Acute oral LD_50_ of demethylated curcuminoid composition was > 5000 mg/kg in rats and no toxicity was found in the dose-dependent 90-day subchronic toxicity study [381]
			• Curcuminoid-essential oil complex showed no toxicity signs at 5000 mg/kg in acute studies nor after repeated administration for 90 days at 1000 mg/kg in rats [382]
			• LD_50_ of aqueous, methanol, and n-hexane root extracts was > 5000 mg/kg oral single dose in rats, and no toxicity was found < 1000 mg/kg after 4 weeks of administration [383]
			• Aqueous extract was not toxic in mice in a subacute study [384]

32	*Cymbopogon citratus* (DC.) Stapf	Lemon grass	• Essential oil had an LD_50_ of 3500 mg/kg single oral dose in mice, and no toxicity was noted in repeated dose of up to 500 mg/kg taken orally for 21 days [356]; LD_50_ of 8 g/kg p.o. single dose was noted in mice, and in subacute studies, organ toxicity was noted for repeated doses > 8 g/kg p.o. for 28 days [385]; LD_50_ was ∼ 3000 mg/kg single oral dose in mice [386]
			• Aqueous extract had LD_50_ ≥ 5000 mg/kg oral dose in mice [387]
			• LD_50_ of oil in rats was 3250 mg/kg p.o., and mortality was only recorded at oral doses > 1500 mg/kg during 14-day administration [388]

33	*Cyperus esculentus* L.	Tiger nut, yellow nutsedge	• Methanol extract was not toxic with LC50 ≥ 1000 μg/mL in brine shrimp; however, the hexane extract was toxic [389]
			• Hydromethanolic extract was ≥ 5000 mg/kg p.o. in mice [390]
			• Aqueous extract had an LD_50_ ≥ 5000 mg/kg oral dose in rats, and no toxicity was noticed at repeated oral doses up to 1000 mg/kg after 28 days [391]; acute toxicity in mice indicated an LD_50_ of 3800 mg/kg p.o. [392]
			• Aqueous tuber extract was not toxic when orally administered in rats [393]

34	*Detarium senegalensis* J.F.Gmel.	Sweet datar, wild cassia	• Aqueous stem bark extract had LD_50_ ≥ 5000 mg/kg oral dose in rats [394]
			• Meal supplementation with 10%, 20%, and 30% of the plant showed no toxicity in rats after 6 weeks of administration [395]

35	*Fragaria vesca* L.	Strawberry	• Ethanol fruit and whole extracts had an LD_50_ ≥ 2000 mg/kg p.o. in rodents [396, 397]
			• Alcohol and aqueous extracts showed no signs of toxicity at 4000 mg/kg i.p. in rodents [398]

36	*Garcinia kola* Heckel.	Bitter kola	• Aqueous seed extract at 25–100 mg/kg/day caused functional toxicity to organs in rats after 7 days of administration [399]
			• Methanol seed extract had LD_50_ > 5000 mg/kg oral dose in mice [400]
			• Ethanol seed extract had LD_50_ > 5000 mg/kg oral dose in rats, and the administration of 100 and 200 mg/kg of the extract did not result in changes in biochemical or hematological parameters after 6 weeks [401, 402]
			• Methanol stem bark extract had LD_50_ of 358 mg/kg oral dose in mice [403]
			• Methanol seed extract had an LC_50_ of 415.72 mg/L in fish after 96 h [292]

37	*Glycine max* (L.) Merr.	Soybeans	• Soyabean oil showed low toxicity at 250 and 500 mg/kg/day po in rats after 30 days [404]
			• LD_50_ value of ethanol extract of Detam-1 soybean above 2000 mg/kg po was noted in mice [405]
			• The LD_50_ of seed extracts was 0.20 mg/g i.p and above for different cultivars in rats [406]
			• LD50 of black soybean was > 2500 mg/kg po in mice and rats, and no adverse effect was found at 5.0% in the diet in chronic studies that lasted for 26 weeks [407]

38	*Gnetum africanum* Welw.	Okazi, Afang	• Methanol leaf extract had LD_50_ ≥ 5 g/kg p.o. single dose, with NOAEL of 40 mg/kg after 90-day administration in rats [408, 409]; LD_50_ ≥ 3000 mg/kg p.o. in rats, and no adverse hematological effects were noted at 200 mg/kg and above after 30 days [410]
			• Aqueous leaf extract displayed low toxicity on testis at 500 mg/kg orally after 3 weeks in rats [411]; no adverse effect was noted on pancreas after oral administration of 0.3–0.5 mL/kg/day for 21 days [412]

39	*Gongronema latifolium* Benth.	Utazi, Bush buck	• Methanol leaf extract had an LC_50_ of 688.66 mg/L in fish after 96 h [292]; LD_50_ was 1581 mg/kg when administered i.p. to mice [413]
			• Ethanol leaf extract had LD_50_ > 5 g/kg p.o. and 1500 mg/kg i.p. in mice [414]; LD_50_ > 3000 mg/kg oral dose in rats [415]; signs of toxicity were noted after repeated doses of 1000 mg/kg during a 90-day oral toxicity study [416]
			• Fruit extract had an LD_50_ > 5000 mg/kg bw for both mice and rats [417]
			• No toxicity was recorded after the oral administration of n-butanol leaf fraction at 5000 mg/kg p.o. in rats [418]

40	*Guiera senegalensis* J.F.Gmel.	Moshi medicine	• Aqueous leaf extract LD_50_ was ≥ 5000 mg/kg oral dose in rats [419, 420]; no toxicity was observed in rats administered 2000 mg/kg p.o. single dose, but mild toxicity was noted for 200 mg/kg/day p.o. after 28 days in rats [421]; LD_50_ of 1264.49 mg/kg p.o. and 316.22 mg/kg i.p. was noted in mice [422]; no toxicity to brine shrimp was noted at concentrations < 1000 μg [423]; no significant toxicity was noted when administered at 2 g/kg to rats for 6 months [424]
			• Methanol root extract LD_50_ was ≥ 5000 mg/kg oral dose in rats [425]
			• LD_50_ of methanol stem extract was ≥ 2000 mg/kg oral dose in rodents [426, 427]
			• LD_50_ of leaf aqueous extract was calculated as 500 mg/kg i.p. in rats [428]
			• Ethyl acetate root extract had an LD_50_ of 1160 mg/kg p.o. for single dose in rats and that for repeated doses was 400 mg/kg after 28-day administration, with no changes in biochemical and hematological parameters noted [429, 430]
			• Aqueous root extract had LD_50_ > 5000 mg/kg p.o. in both mice and rats [431]
			• Aqueous gall extract indicated cytotoxicity at 90 μg/mL in chicken embryo skin cells, but no toxicity was recorded when 100 mg/kg/day p.o. was given to chickens at 500 and 1000 mg/kg/day [432]
			• Ethanol leaf extract exhibited no toxicity to mice at doses up to 400 mg/kg p.o. [433]
			• Methanol and hexane leaf extracts had no toxic signs at 5000 mg/kg p.o. in rats; however, mild toxicity was noted after 4 weeks in liver function and electrolyte levels at 500 and 1000 mg/kg/day [434]
			• Methanol leaf extract had LD_50_ ≥ 5 g/kg p.o. in rats with no toxicity after intake of 1 g/kg/day p.o. for 60 days [435]; resulted in death and organ enlargement at 500 and 1000 mg/kg when injected intramuscularly in rats [436]
			• Methanol gall extract had an LD_50_ > 2 g/kg p.o. in rats [437]

41	*Heinsia crinita* (Afzel.) G.Taylor	Bush apple	• No toxicity was found for the root bark extract in acute toxicity study (LD_50_ > 5 g/kg p.o.), nor in subacute test at < 800 mg/kg/day when orally administered for 4 weeks [438]

42	*Hibiscus sabdariffa* L.	Roselle	• Ethanol seed extract had an LD_50_ ≥ 5 g/kg p.o. in albino rats [439, 440]
			• Aqueous extract showed no toxic or mutagenic activity when rats received 400 mg/kg/day i.p. for 15 days [441]
			• Hot water calyx extract showed no acute toxicity (LD_50_ > 5 g/kg p.o.) or chronic toxicity when ingested at doses < 200 mg/kg for 270 days in rats [442]
			• Ethanol calyx extract had an LD_50_ above 5000 mg/kg p.o. in rats [443]; administration of 200 mg/kg p.o. for 35 days led to toxic signs from rat kidneys [444]
			• Chloroform, petroleum ether, ethanol, and water extracts of calyxes were not toxic at 2 g/kg p.o. in mice [445]
			• Calyx extract infusion had an LD_50_ > 5 g/kg p.o. and continuous infusion of extract in water showed no toxicity at doses < 1 g/kg in rats [446]
			• Fermented seed incorporated in the diet at 10%, 15%, and 20% showed enterohepato nephrotoxicity in rats [447]

43	*Ipomoea batatas* (L.) Poir.	Sweet potato	• Ethanol leaf extract indicated an LD_50_ > 5 g/kg p.o. in rats; however, administration of doses > 1 g/kg produced toxicity in vital organs after 28 days [448]
			• Ethyl acetate and methanol tuber extracts had an LD_50_ > 4000 mg/kg p.o. in rats [449, 450]; in vitro results showed nontoxic activity in rabbit articular chondrocytes at concentrations < 1000 μg/mL [450]
			• Peel tuber extract via topical administration was not toxic < 2000 mg/kg in rats [451]
			• Aqueous tuber extract was orally nontoxic < 2000 mg/kg in rats [452]
			• No toxicity was observed at 5000 μg/mL of 20% ethanol tuber extract in 3T3-L1 fat cells [154]
			• Ethanol leaf extract at 200 μg/mL revealed moderate signs of cytotoxicity on BV2 microglia cells [453]

44	*Irvingia gabonensis* Baill.	Wild mango	• LD_50_ of aqueous seed extract was ≥ 5 g/kg oral single dose in rats [454]
			• Oral lethal dose of the ethanol leaf and stem bark extracts was ≥ 5000 mg/kg in rats [455, 456]
			• Aqueous extracts of stem bark and leaf displayed no toxic effect on the heart, liver, spleen, and kidney, except for testes in male rats administered oral doses ≥ 1000 mg/kg [457]
			• No genotoxicity was noted in vitro and the NOAEL was 2500 mg/kg after 90 days administration in rats [458]

45	*Khaya senegalensis* (Desv.) A.Juss.	African mahogany	• LD_50_ ≥ 3000 mg/kg single oral dose of aqueous leaf extract in rats was reported; repeated doses up to 3000 mg/kg p.o. for 28 days did not produce toxicity [459]
			• LD_50_ of water stem bark extract was 1778 mg/kg intraperitoneally in mice [460]; LC_50_ was 2.7 mg/mL on *Artemia salina* larvae, while on rats, the LD_50_ was ≥ 2 g/kg oral single dose [461]; LD_50_ was 4.2 g/kg in rats, but oral administration at doses up to 16.28 mg/kg once daily for 3 weeks caused hepatocellular toxicity in rats [462]
			• Aqueous stem bark extract LD_50_ was ≥ 5 g/kg p.o in rats; however, mild toxicity on electrolyte levels, liver, and kidney function was detected at doses ≥ 1600 mg/kg/day after 28 days [463]
			• Leaves added to rabbit diet at 35% and 65% caused mild adverse effect on liver and kidney function in a dose-dependent manner [464]

46	*Luffa aegyptiaca* Mill.	Luffa	• Ethanol fruit extract did not result in any mortality when administered orally at 2000 mg/kg in rats [465]

47	*Lycopersicon esculentum* Mill.	Tomato	• Leaf extract was nontoxic at 5 g/kg oral single dose, whereas repeated oral doses ≥ 1 g/kg after 28 days cause mild toxicity [466]; acute and residual toxicity was noted against *Drosophila melanogaster* wild-type flies of different stages [467]
			• Oral administration of tomato pomace single dose of 1000 and 5000 mg/kg for 2 weeks did not result in toxicity in rats [468]

48	*Mentha pulegium* L.	Mint leaf	• Methanol extract had antigenotoxic effects on human lymphocyte culture [469]
			• Aqueous extract altered reproductive performance and induced fetotoxicity of female rats at > 2.0 g/kg after 20 days of administration, LD_50_ was ≥ 5 g/kg [470]
			• Essential oil showed potential toxicity with LD_50_ of 14 μg/mL for 3 human cancer cell lines [471]; LC_50_ value was 0.09 μL/mL air for *C. maculatus* [472]

49	*Momordica balsamina* L.	Balsam apple, African cucumber	• No report found

50	*Momordica charantia* L.	Bitter melon	• Ethanol extract is considered safe for consumption at < 2000 mg/kg [473]; LD_50_ was 200 mg/L and 50 mg/L after 24 and 96 h exposure period, respectively, in fishes [474]
			• Methanol leaf extract was tolerated by the rats at 200 mg/kg in mice [475]
			• Hydroalcoholic extract had an oral LD_50_ > 5 g/kg in mice [476]; LD50 > 2000 mg/kg oral single dose in rats [477, 478]
			• Alkaloid rich-fractions indicated toxicity at 600 ppm in zebra fish embryos [479]
			• Aqueous extract had LC_50_ between 144 and 251 *μ*g/mL in zebrafish embryos [480]
			• LD_50_ of fruit alcoholic extract was 362.34 mg/100 g in mice [481]
			• Ethanol seed extracts at 800 mg/kg/day after 42 days of administration caused toxicity in reproductive organs resulting in infertility in male rats [482]
			• No toxicity was observed at 2000 mg/kg for ethanol and aqueous extracts when administered to rats [483]

51	*Moringa oleifera* Lam.	Moringa, drumstick tree, miracle tree	• LC_50_ after 96 h of methanol leaf extract was 413.85 mg/L in fish [292]
			• Aqueous leaf extract showed no toxicity at 10 g/kg p.o in mice, nor chronic toxicity in male rats; however, in female rats, toxicity was noted at doses from 100–1000 mg/kg after 6 months [484]; LD_50_ of 1 g/kg p.o. single dose was noted, and no toxicity was noted after repeated doses of up to 80 mg/kg in rodents [485]; LD_50_ was calculated as 1585 mg/kg i.p. and ≥ 6400 mg/kg p.o. in rats, and no toxicity was noted at 1500 mg/kg/day orally after 60 days of administration [486]
			• Oral LD_50_ was ≥ 2000 mg/kg for dried leaf powder in rats [487]; oral administration at 1000 mg/kg daily showed no changes in clinical signs nor gross pathology after 90-day administration in rats [488]
			• Oral ingestion of methanol extracts of leaf and seed by rats indicated mild toxicity in vital organs at > 1000 mg/kg after 28 days [489]
			• Ethyl acetate leaf fraction showed mild toxicity to vital organs at doses up to 400 mg/kg p.o. when administered to chicks after 28 days [490]
			• LD_50_ of the leaf aqueous-methanol extract was > 2 g/kg oral dose in rats [491]
			• LD_50_ of methanol leaf extract was 6.61 g/kg for rats and 26.04 g/kg for rabbits [492]; LD_50_ was 5.47 g/kg p.o. in mice, while subacute studies showed no toxicity at 2.5 g/kg [493]
			• Leaf extract showed no acute toxicity (LD_50_ > 5 g/kg p.o.) but potential mild subacute toxicity at 1 g/kg p.o. in rats after 14 days [494]
			• LD_50_ of the methanol seed extract was > 3.8 g/kg p.o. in rats, while subacute administration of 1.6 g/kg/day for 3 weeks revealed slight toxicity on liver enzymes [495]
			• Lethal dose of the ethanol leaf extract in mice was > 2600 mg/kg but < 5000 mg/kg [496]; lethal dose of ethanol leaf extract was 6616.67 mg/kg i.p. for rats and 26,043.67 mg/kg i.p. for rabbits [492]

52	*Mucuna flagellipes* Hook.f.	Ukpo.	• LD_50_ of ethanol seed extract was 1250 mg/kg oral dose in mice [497]

53	*Murraya koenigii* (L.) Spreng.	Curry leaf	• Administration of ethanol leaf extract for 28 days was safe at doses below 900 mg/kg oral dose in rats [498]
			• LDH of HepG2 cells peaked at 15.74% of methanol curry leaf extracts indicating low acute cytotoxicity [499]
			• Methanol leaf extract indicated toxicity and mortality at doses > 200 mg/kg/day in rats during a 10-week study [500]; no toxic effect was observed at doses < 9000 mg/kg p.o. in mice [501]
			• Lethal dose of ethanol leaf extract was > 5 g/kg oral single dose in mice [502]

54	*Myristica fragrans* Houtt.	Nutmeg	• Ethanol mace extract was safe below 500 mg/kg single oral dose in rats [503]
			• Oral intake of 400 mg/kg and above of aqueous extract indicated possible toxicity to organs after period of 28 days [504]
			• Acetone mace extract did not cause any apparent toxicity in rats at 4 g/kg p.o. after 7 days [505]
			• LD_50_ of crude alkaloids was 5.1 g/kg oral dose in mice [506]
			• LC_50_ of the essential oil was 5434.78 ± 23.2 μg/mL in brine shrimp indicating noncytotoxicity (LC_50_ > 1000 μg/mL) [507]
			• LD_50_ in mice was 4000 mg/kg oral dose of 50% ethanol extract [508]

55	*Ocimum basilicum* L.	Basil	• Essential oil of leaf had LC_50_ of 73.45 ppm on *Culex pipiens* larvae [509]
			• Hydroalcoholic leaf extract had an LD_50_ of 35.44 μg/mL in leukocytes [510]; it was nontoxic at 2 g/kg single oral dose in rats, while in subchronic study, no adverse effects were observed on serum parameters with the exception to hematological changes in male and female rats [511]; LC_50_ of 9.92 μg/mL in *Artemia salina* was noted [270]
			• Extract of leaves at doses up to 1500 mg/kg/day p.o. was not toxic after 29-day administration to mice [512]

56	*Ocimum gratissimum* L.	Scent leaf, fever plant, fever bush	• Aqueous leaf extract was not toxic at 4000 mg/kg oral dose in rats [513]; LD_50_ of 4.5 g/kg single oral dose was noted although it was nontoxic up to 400 mg/kg/day in rats for 14-day administration [514]
			• Leaf fractions of ethyl acetate and petroleum ether induced dose-dependent hepatorenal toxicity at oral doses ≥ 400 mg/kg in rodents [515]
			• Rats administered < 500 mg/kg of aqueous extract for 21 days showed no mortality, but mild toxicity to the kidneys [516]
			• Butanol fraction from crude methanol leaf extract was nontoxic at doses up to 400 mg/kg p.o. to male reproductive system but exhibited fertility-enhancing effects [517]
			• Aqueous leaf extract exerted some functional damage to the kidney and liver at doses above 0.4 g/kg after 4 weeks in rabbits [518]
			• LD_50_ of oil was 0.27 g/kg i.p and 1.41 g/kg p.o in mice and 0.43 g/kg i.p and 2.29 g/kg p.o in rats, respectively, and repeated daily doses of > 133 mg/kg i.p. caused toxicity in the rats [519]
			• Ethanol leaf extract at 300 mg/kg p.o. demonstrated no significant toxicity in biochemical and histopathological parameters in rats [520]

57	*Persea americana* Mill.	Avocado pear	• LD_50_ of fruit was ≥ 5 g/kg p.o. in rats, and the administration of 10 g/kg for 8 weeks revealed no toxicity in rats [521]
			• LD_50_ of ethanol seed extract was ≥ 5 g/kg p.o. and 2.25 g/kg i.p. in mice [522]
			• Ethanol peel extract had no toxicological effects, and LC_50_ was 204.95 mg/mL against *Artemia. salina* [523]
			• Ethanol seed extract had LD_50_ of 1200.75 mg/kg p.o. in mice; also, in vivo genotoxic activity in peripheral blood cells was not observed [524]
			• Maximum tolerated dose of aqueous seed extract was 10 g/kg p.o., and no toxicity at 2.5 g/kg/day p.o. per day after 4 consecutive weeks of administration was noted in rats [525]
			• Oral LD_50_ for seed flour was 1767 mg/kg in mice [526]
			• Aqueous leaf extract showed signs of hepatotoxicity in rats at 750 mg/kg p.o. after 28 days of administration [527]
			• LD_50_ of ether extract of seed was 751.6 ± 98.6 mg/kg i.p. in rats while a daily administration of 75 and 150 mg/kg i.p. for 14 days did not cause significant toxicity in subacute study [528]

58	*Piper guineense* Schumach. & Thonn.	Uziza seeds, Ashanti pepper, Benin pepper	• LC_50_ of methanol leaf extract was 205.35 mg/L in fish after 96 h [292]; extract was well tolerated at a dose of 5000 mg/kg p.o. in rats [529]
			• Ethanol leaf extract had LD_50_ > 3000 mg/kg p.o. in rats [415]; extract was safe at a dose of 2 g/kg p.o. in mice [530]
			• Seed powder at 5% and 10% was toxic to maize weevil [531]
			• LD_50_ values of the plant fruits were 85.1 mg/kg i.v, 224 mg/kg i.p., and 1122 mg/kg p.o. in rats [532]
			• Estimated LC_50_ values of ethanol and hot water fruit extracts were 0.10 + 0.04 mg/L and 5.0 + 1.4 mg/L in adult *Biomphalaria pfeifferi* [533]
			• Essential oil of fruits had LD_50_ of 693 mg/kg i.p. and 1265 p.o. in mice [534]
			• Aqueous fruit extract had an LC_50_ of 3.1 mg/cm^3^ after 96 h in fish [535]

59	*Piper nigrum* L.	Black pepper	• LD_50_ of fruit was > 5 g/kg p.o. in rats, but subacute toxicity at 0.62 mL revealed architectural distortions of the kidneys, liver, and testes [536]
			• LD_50_ of aqueous fruit extract was > 5000 mg/kg p.o. in rats; also, repeated doses of 1200 mg/kg p.o. for 90 days did not produce any significant damage to the internal organs in subchronic studies [341]; no toxicity was noted up to 5 g/kg p.o. in mice [537, 538]
			• Single oral dose of piperine-free extract revealed an LD_50_ > 5 g/kg in mice [539]
			• Intraperitoneal LD_50_ was 19.36 mg/kg in mice and > 200 mg/kg in rats for piperine, hexane fruit extract, and ethanol fruit extract [540]
			• No signs of toxicity were noted at a dose of 200–400 mg/kg b.w. of piperine in rats [196]

60	*Prosopis africana* (Guill. & Perr.) Taub.	False locust	• In acute toxicity test, LD50 of 774 mg/kg i.p. of methanol extract of *P. africana* stem bark was established in mice [541]
			• LD50 of the methanol seed extract was ≥ 5000 mg/kg p.o. in rats [542]
			• Oral LD_50_ of the methanol stem bark extract was 3807.9 mg/kg in mice and > 5000 mg/kg in rats [543]
			• Oral LD_50_ of the aqueous and hydroethanolic leaf extracts was estimated as 5000 mg/kg while 1000 mg/kg was estimated for alkaloid-enriched fraction of aqueous extract in mice [544]

61	*Psidium guajava* L.	Guava leaves	• Aqueous bark and leaf extracts had an LC_50_ of 480.14 and 949.13 μg/mL against *Artemia salina* [545]
			• The 10–50 mg/100 g single oral dose of the aqueous extract showed no signs of toxicity in rats [546]
			• No toxicity was observed at 2000 mg/kg p.o. of 50% methanol–water leaf extract in rats [547]
			• Ethanol fruit extract was not toxic (LD_50_ > 5 g/kg p.o.) to the liver and kidney in mice [548]
			• NOAEL estimated of white, red, and pink guava leaf extract was 50–5000 mg/kg p.o. on vital organs in rats after14 days of administration [549]
			• LD_50_ of the methanol bark extract was ≥ 5000 mg/kg, but at doses ≥ 500 mg/kg for 28 days, it exhibited organ toxicity in rats [550]
			• LD_50_ of root ethanol extract was 1352 mg/kg p.o. in rats, while subchronic studies showed that administration of 150–1200 mg/kg daily of the extract for 90 days was toxic to rats [551]

62	*Pterocarpus mildbraedii* Harms	Ora, Oha	• LD_50_ values of subfractions of extract were ≥ 2000 mg/kg p.o. in rats [552]
			• LD_50_ values of ethanol and aqueous leaf extracts were 1258 and 1778 mg/kg i.p. in rats while repeated daily doses of 400 mg/kg i.p. to rats for 28 days showed no adverse effects on the vital organs and hematological parameters [553, 554]

63	*Senna occidentalis* (L.) Link	Coffee senna	• Seed supplementation in rat feed at 1%–4% resulted in toxicity to the thymus, lymphoid organs, and hematopoietic systems in the rats after 14 days [555]; death and damage to liver and heart were found in rats fed 4% after 30 days [556]
			• Aqueous leaf extract showed no acute toxicity (LD_50_ > 5000 mg/kg) in rats; also, daily doses of 3000 mg/kg for 28 days had no significant toxicity on the kidneys of rats [557]; LD_50_ was ≥ 3000 mg/kg p.o. in rats [558]
			• The inclusion of 0.2% of external and internal tegument of seeds was toxic for laying hens after 42 weeks of administration [559]

64	*Spinacia oleracea* L.	Spinach	• No toxicity was observed at 2 g/kg oral single dose of the ethanol extract in mice [560]
			• Lethal dose of ethanol and aqueous leaf extracts was 2000 mg/kg p.o. in rats [561]

65	*Tamarindus indica* L.	Tamarind	• LD_50_ of ethanol and aqueous pulp extract was ≥ 5000 mg/kg p.o. in rats [562, 563]; repeated doses < 4500 mg/kg of aqueous pulp extract for 28 days did not indicate significant toxicity in the rats [562]
			• Methanol fruit extract showed no signs of toxicity at repeated oral doses < 2000 mg/kg in rats after 28 days [564]
			• Oral administration of a single dose (2000 mg/mL) of the leaf fluid extract showed signs of toxicity in rats [565]
			• Tamarind leaf ethanol extract had LD_50_ > 2000 mg/kg in rats [566]
			• Ethanol stem bark extract had LD_50_ values of 1542 μg/mL and 2720 μg/mL in brine shrimps and chicken embryos, respectively [567]
			• Chronic toxicity study indicated the aqueous pulp extract to be safe to < 1 g/kg p.o. [568]

66	*Tetrapleura tetraptera* (Schumach. & Thonn.) Taub.	Aiden pod	• Ethanol pod extract indicated toxicity in vital organs when orally administered at doses between 50 and 200 mg/kg in mice for 42 days [569]
			• Methanol extracts of various plant parts had LC_50_ values from 1.50 to 3.16 mg/L in mollusc [570]
			• Methanol fruit extract was not toxic < 5000 mg/kg p.o. in rats [571]
			• Oil extract of fruits showed molluscicidal activity with LC_50_ of 0.43 mg/L after 48 h exposure in freshwater snails [572]
			• LC_50_ of leaf powder was 1.60 g/L in *C. gariepinus* juveniles after 96 h exposure and at this dose, the hematological parameters were altered [573]
			• No mortality or any significant gross pathological changes were observed < 2000 mg/kg for the aqueous fruit extract [574]; LD_50_ = 244.94 mg/kg in mice when administered i.p. which was associated with changes in hematological parameters [575]

67	*Vernonia amygdalina* Delile	Bitter leaf	• LD_50_ of aqueous leaf extract was ≥ 5000 mg/kg p.o. in rats [576, 577]; it did not induce toxicity at doses up to 1000 mg/kg/day p.o. in rats after 28 days [578]; LC_50_ was 1.49 ± 0.19 mg/mL in brine shrimp [579]
			• Ethanol root extract had an LD_50_ > 2400 mg/kg [580]
			• Ethanol leaf extract at oral doses > 300 mg/kg/day demonstrated testicular toxicity in rats after 56 days [581] and hepatotoxicity after 28 days [582]

68	*Vigna subterranea* (L.) Verdc.	Bambara groundnut	• No report found

69	*Zingiber officinale* Roscoe	Ginger	• Oil was nontoxic after repeated oral administrations of up to 500 mg/kg/day to rats for 13 weeks [583]
			• Aqueous extract indicated an LD_50_ > 2000 mg/kg p.o. in broiler chickens [584]
			• Fixed oil-induced oxidative damage, damage to the organs, and cellular toxicity were noted at doses > 0.02 mL/kg/day after 60 days [585]
			• LD_50_ of volatile oil was 8.051 ± 1.254 and 12.99 ± 1.201 mL/kg in mice and rats, respectively, and subchronic studies did not show any significant changes in the hematology results in mice [586]
			• Single oral dose of 2.5 g/kg was toxic and resulted in severe hypotension and bradycardia [587]
			• Ethanol root extract was found safe when administered for 35 days p.o. at 2000 mg/kg/day, but higher doses were toxic [588]
			• Methanol extracts up to 600 mg/kg oral daily for 4 weeks were considered safe in rats [589]

70	*Ziziphus jujube* Mill.	Jujube fruit	• Ethanol leaf extract resulted in no mortality up to 5000 mg/kg p.o. in rats [590, 591]
			• Aqueous root had LD_50_ ≥ 2500 mg/kg in rats when administered p.o. [592]
			• Jujube oil and petroleum ether leaf extracts were toxic to *Culex pipi*ens with LC_50_ values of 0.30% and 0.53%, respectively [593]
			• Aqueous extract exhibited cytotoxicity (IC_50_: 1.671 mg/mL) in C643 cells [594]

*Note:* CC_50_, half cytotoxic concentration; ED_50_, median effective dose; HepG2, human hepatoma; IC_50_, half maximal inhibitory concentration; LC_50_, median lethal concentration; LD_50_, median lethal dose; p.o., per os (oral).

Abbreviations: BMI, body mass index; bw, body weight; DIO, diet-induced obesity; HCD, high-carbohydrate diet; HFD, high-fat diet; i.m., intramuscular; i.p., intraperitoneal; i.v., intravenous; NOAEL, no observed adverse effect level; NPD, normal pellet diet.

## Data Availability

The data supporting the results of this study have been included in the research article.

## Nomenclature

AMP: Adenosine monophosphate
AMPK: Adenosine monophosphate–activated protein kinase
BMI: Body mass index
C/EBP: CCAAT/enhancer binding proteins
CB1: Cannabinoid receptor type 1
CoA: Coenzyme A
DIO: Diet-induced obesity
ED_50_: Median effective dose
FAS: Fatty acid synthase
GLP-1: Glucagon-like peptide-1
HCD: High-carbohydrate diet
HepG2: Human hepatoma
HFD: High-fat diet
HMG-CoA: β-Hydroxy β-methylglutaryl-CoA
HSL: Hormone-sensitive lipase
IC_50_: Half maximal inhibitory concentration
LC_50_: Median lethal concentration
LD_50_: Median lethal dose
LDH: Lactate dehydrogenase
LLC-MK2: Rhesus monkey kidney epithelial cells
LPL: Lipoprotein lipase
MAPK: Mitogen-activated protein kinase
mRNA: Messenger RNA
MSG: Monosodium glutamate
NOAEL: No observed adverse effect level
NPD: Normal pellet diet
PI3-K: Phosphoinositide 3-kinases
PPARγ: Peroxisome proliferator–activated receptor-γ
RBP-4: Retinol binding protein 4
SREBP-1: Sterol regulatory element–binding transcription factor 1
TNF: Tumor necrosis factor
TRPV-1: Transient receptor potential cation channel subfamily V member 1
UCP2: Uncoupling protein 2
WAT: White adipose tissue
